# Influence of White and Gray Matter Connections on Endogenous Human Cortical Oscillations

**DOI:** 10.3389/fnhum.2016.00330

**Published:** 2016-06-28

**Authors:** Ammar H. Hawasli, DoHyun Kim, Noah M. Ledbetter, Sonika Dahiya, Dennis L. Barbour, Eric C. Leuthardt

**Affiliations:** ^1^Department of Neurological Surgery, Washington University School of MedicineSaint Louis, MO, USA; ^2^Department of Biomedical Engineering, Washington University School of MedicineSaint Louis, MO, USA; ^3^Department of Pathology and Immunology, Washington University School of MedicineSaint Louis, MO, USA

**Keywords:** human neuroscience, neurophysiology, electrocorticography, cortical oscillations, cortical physiology

## Abstract

Brain oscillations reflect changes in electrical potentials summated across neuronal populations. Low- and high-frequency rhythms have different modulation patterns. Slower rhythms are spatially broad, while faster rhythms are more local. From this observation, we hypothesized that low- and high-frequency oscillations reflect white- and gray-matter communications, respectively, and synchronization between low-frequency phase with high-frequency amplitude represents a mechanism enabling distributed brain-networks to coordinate local processing. Testing this common understanding, we selectively disrupted white or gray matter connections to human cortex while recording surface field potentials. Counter to our original hypotheses, we found that cortex consists of independent oscillatory-units (IOUs) that maintain their own complex endogenous rhythm structure. IOUs are differentially modulated by white and gray matter connections. White-matter connections maintain topographical anatomic heterogeneity (i.e., separable processing in cortical space) and gray-matter connections segregate cortical synchronization patterns (i.e., separable temporal processing through phase-power coupling). Modulation of distinct oscillatory modules enables the functional diversity necessary for complex processing in the human brain.

## Introduction

Rhythmic brain activity is routinely measured for neuroscientific inquiry and for clinical evaluation of disease. This oscillatory activity reflects changes in cortical potentials caused by a summation of firing in neuronal populations. Systematic changes in cortical oscillations accurately reflect cognitive operations and correlate with activity in neuronal populations (Pfurtscheller and Aranibar, 1977; Miller et al., 2007; Logothetis, 2008; Manning et al., 2009; Gaona et al., 2011). A long history of research has been dedicated to understanding the anatomical substrates underlying cortical oscillations. Several lines of evidence support cortical and subcortical roles in the generation, modulation, and physiological relevance of oscillations (Kristiansen and Courtois, 1949; Andersen and Sears, 1964; Szerb, 1967; Andersen and Andersson, 1968; Ulmar et al., 1975; Llinas, 1988; Pedley and Traub, 1990; Llinas et al., 1991, 1999; Timofeev and Steriade, 1996; Bollimunta et al., 2008). Despite the ubiquity of human cortical oscillations in research and biomedicine, the underlying circuits, origins and physiological relevance of these rhythms remain incompletely characterized.

To better understand the macroscale white matter and gray matter circuits underlying human cortical oscillations, we developed a lesion model in humans undergoing the resection of normal cortical tissue as a necessary and expected part of their surgical procedure. We tested three fundamental hypotheses on the neural circuitry underlying cortical rhythms by selectively disrupting white or gray matter connections (reflecting long- or short-range circuits, respectively) to a region of neocortex and recording local field potentials. First, we tested the hypothesis that low- and mid-frequency rhythms, notably μ/β rhythms between 8 and 23 Hz, predominantly arise from white-matter circuitry modulating local cortical activity (Pfurtscheller, 1981; Crone et al., 1998b; Steriade and Timofeev, 2003; Buzsaki and Draguhn, 2004). Therefore, transecting white matter beneath a cortical site should preferentially affect these low- and mid-range frequency rhythms. Second, low- and mid-range frequency rhythms are thought to be associated with broad topographic distributions, while higher frequency γ rhythms (>70 Hz) are thought to represent more focal cortical activations (Crone et al., 1998a; Miller et al., 2007). Therefore, selective white matter disconnection of cortex underneath one region of cortex would be expected to reduce measures of functional connectivity, such as mutual information, in the low-/mid-frequency bands between an experimental site and adjacent cortex. This would also be expected if low-frequency information was driven by a separate source (e.g., the thalamus) because loss of white matter inputs into the experimental region would abolish inputs form a distant third source. Gray matter separation between these two cortices would be expected to reduce the shared information in high-frequency γ-range. And third, when considering low- to high-frequency interactions, low-frequency band phase to high- γ-range amplitude coupling has been thought to represent a mechanism for enabling communication between distant neuronal populations (Buzaki, 2006; Canolty et al., 2006). Thus, white matter and gray matter transections should impair or disrupt this coupling.

The results of this study, however, demonstrate unexpected findings that point toward a fundamentally different, cortically-centric, functional organization that explains the brain's oscillatory behavior. We found that selective disruption of white matter connections reduced oscillation power in low-frequencies more than high-frequencies, as expected. Contrary to our hypotheses, however, white matter sectioning increased functional connectivity with adjacent cortical sites (predominantly at low-frequencies) and did not alter phase/amplitude coupling. Disruption of surrounding gray matter connections did not significantly alter oscillatory power but substantively increased cross-frequency phase/amplitude coupling at a synchronized phase. When white matter and gray matter lesions were combined at the same site (i.e., creating functionally-isolated cortex), endogenous oscillations and complex oscillatory cross-frequency relationships (i.e., phase-amplitude coupling) were maintained, or enhanced. These oscillations persisted virtually unaltered for up to 10 min or longer even after cortex was removed. These findings suggest that cortex consists of independent oscillatory-units (IOUs) that maintain their own complex endogenous rhythm structure without the need for external neural input. IOUs are differentially modulated by their white and gray matter connections, where white-matter connections maintain topographical heterogeneity (i.e., separable processing along the cortical surface), and gray-matter connections segregate cortical synchronization patterns (i.e., separable processing in time). Modulation of these distinct oscillatory modules allows for the functional diversity necessary for complex signal processing in the human brain.

### Evaluating human cortical oscillations with selective surgical transections

**Figure 1:**
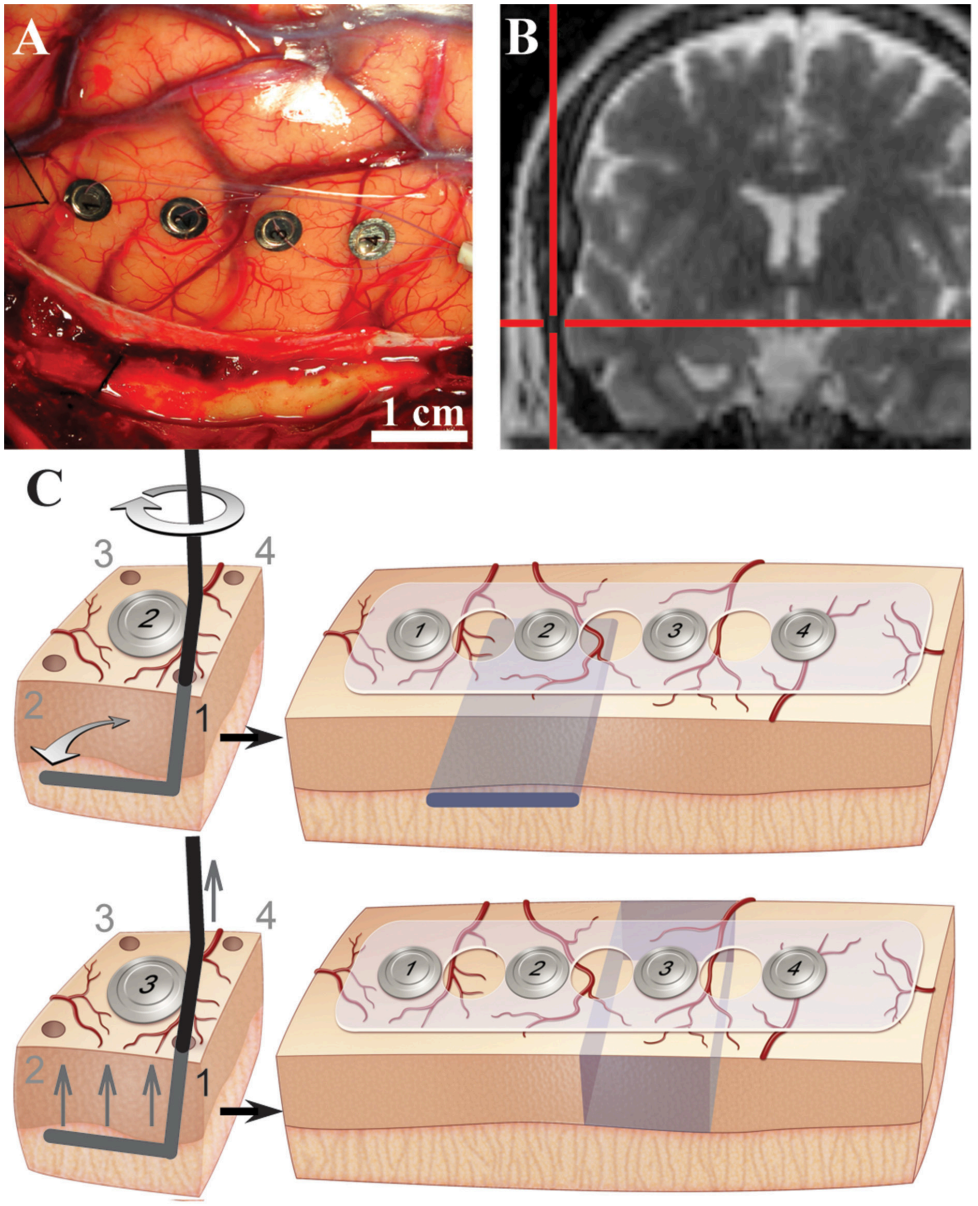
**Evaluation of cortical oscillations with selective white and gray matter disruptions in human cortex. (A)** Intraoperative photograph showing subdural electrocorticography electrodes on human middle temporal gyrus. **(B)** Intraoperative coronal T2-weighted magnetic resonance navigation image showing location of electrodes on middle temporal gyrus. **(C)** Illustrations of subpial white matter disruption at the gray-white junction under electrode #2 (*top*) and selective gray matter disruptions around electrode #3 (*bottom*). Selective lesions were performed through each corner (1–4) as shown. Desired lesions are depicted in blue *(right*).

Neurosurgical access to pathological tissues frequently requires resection of normal neocortex at the surface. This resection of normal tissue is necessary to allow for a direct corridor to access the pathology. In other specific clinical circumstances, surrounding normal tissue is resected along with pathological tissue. To take advantage of this unique opportunity, cortical oscillations were recorded from the sites of planned resection while performing selective white and gray matter transection *in vivo* prior to their removal. The experimental surgical manipulations were performed on cortical regions that were planned for resection and there was no alteration in the standard-of-care of the surgical procedure. Experimental manipulations did not pose any significant additional risk to the human subject. Although valuable, animal data has inherent limitations because interspecies differences have been documented for cortical innervation, anatomy and pathology (Berger et al., 1991; Andersen, 2007; Povysheva et al., 2007, 2008; Engel et al., 2008). Moreover, compared with animal models including non-human primates, the size of the human brain and thickness of human gray matter were advantageous to perform selective and precise gray and white matter transections while maintaining tissue viability. The size of the human gyrus allowed for reliable and selective lesions in the gray and white matter while maintaining the pial blood supply. To define contributions and physiological relevance of white and gray matter connections to human cortical oscillations, mesoscale surface field potentials (i.e., in millimeter dimensions) were recorded intraoperatively by subdural electrocorticography (ECoG) before and after performing selective transections *in vivo* (Figures 1A,B, Supplementary Table 1). Selective and precise disconnection of white matter into overlying cortex was performed through subpial transection of the gray -white junction below an electrode of interest (Figure 1C, *top;* “white”; Supplementary Figure 1). Alternatively, circumferential disruption of gray matter connections from surrounding cortex was performed through selective subpial vertical transections around the electrode of interest (Figure 1C, *bottom;* “gray”).

## Materials and methods

### Subjects and ethics

Ten patients undergoing surgical treatment for intractable epilepsy or brain tumors participated in this study, which received ethical approval by the Washington University School of Medicine Department of Neurosurgery Ethical Review Board Committee and the Human Research and Protection Organization Institutional Review Board at Washington University. Before inclusion, all patients gave written informed consent. Neurosurgical access to pathological tissues often requires resection of normal neocortex at the surface. This resection of normal tissue allows for a direct corridor necessary to access the pathology. In other specific clinical circumstances, surrounding normal tissue is resected along with pathological tissue. Clinical decision making and initial selection of experimental candidates were performed independently by separate individuals (last and first authors, respectively). For each subject, the patient-tailored clinical operative strategy was created prior to and independently of experimental plans; hence, experimental protocols had no bearing or effects on the clinical care of each patient, and the risk of harm to patients was not increased by the experiments. Patients only qualified for the study if they were receiving a corticotomy and resection of cortical tissue (inclusive in the surgical plan, Supplementary Table 1). Magnetic resonance imaging (MRI) and clinical data were collected for each subject. Patients were excluded from the study if they were less than 18 years old, unable to provide informed consent due to impaired mental status, or if the MRI showed dysplastic or absent cortex over intended recording area or abnormality near anticipated recording site. Eight subjects underwent a primary craniotomy for resection of the clinical lesion. Two patients underwent craniotomy for the subdural placement of an electrode array that was then removed (with a second craniotomy) 1 week later for resection of the epileptic foci. See Supplementary Table 1 for demographic and clinical information.

**Figure 2:**
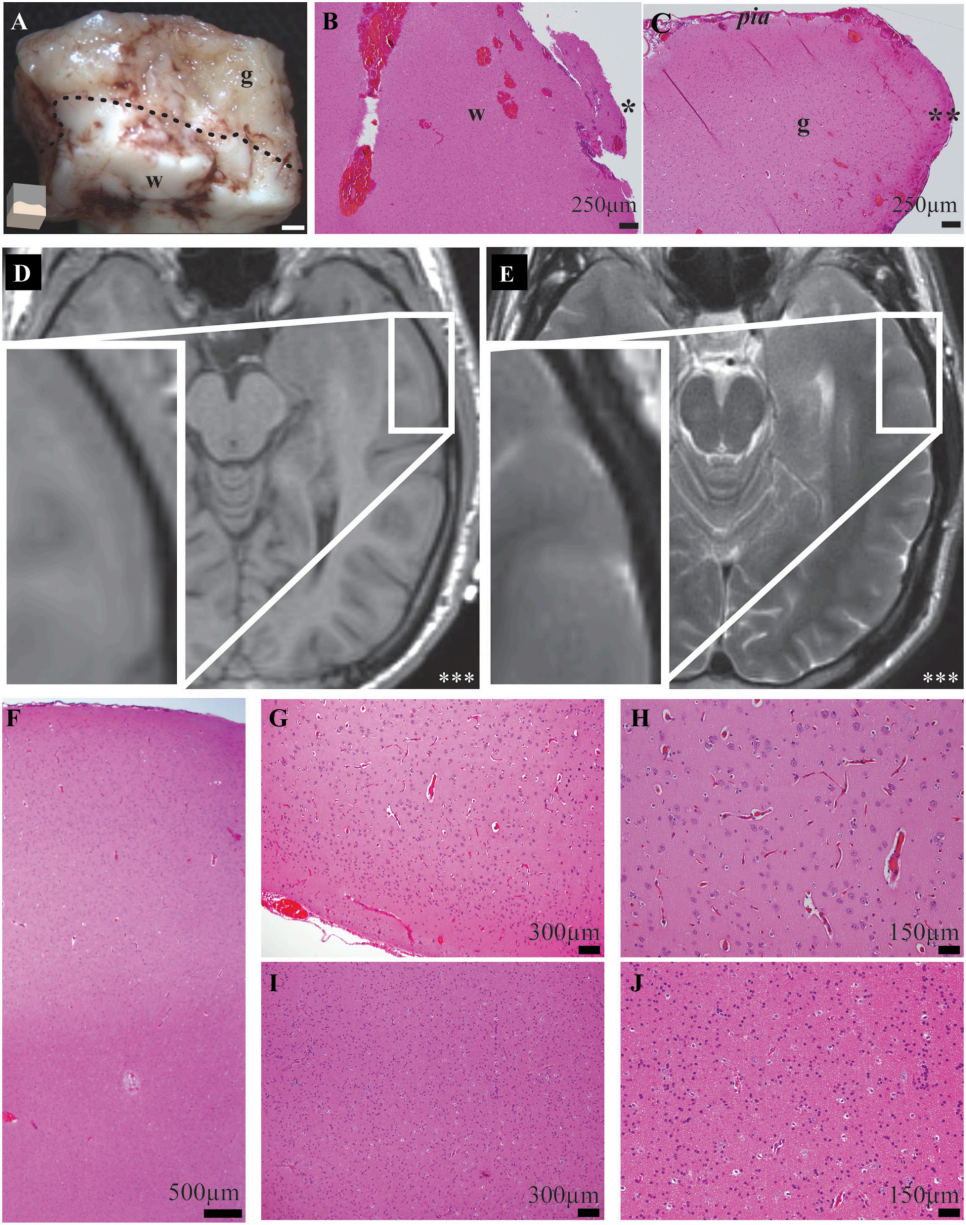
**Exemplar gross, histological, and radiological examinations demonstrate precise and accurate gray and white matter transections and no recording-site tissue abnormalities. (A)** Magnified gross specimen of recording site shows gray (*g*) and white (*w*) matter separated by dotted-line. Illustration (*bottom-left*) shows orientation and white bar represents 1 mm. Microscopy of functionally-disconnected cortex stained with hematoxylin and eosin stain confirms white **(B)** and gray **(C)** matter disruptions. Asterisk indicates edge of white matter transection; Double asterisk indicates edge of gray matter disruption. **(D)** T1- and **(E)** T2-weighted magnetic resonance imaging radiology of recorded neocortex demonstrates no abnormalities. Inset demonstrates magnified recording area and triple-asterisk indicates left side. **(F)** Low magnification microscopy of middle temporal gyrus recording location stained with hematoxylin and eosin fails to demonstrate any causes or sequela of epilepsy or other pathology. Higher magnification microscopy of gray **(G,H)** and white **(I,J)** matter confirm no abnormalities in microscopic laminar architecture and cellular morphology.

Although human recordings offer a unique and valuable opportunity to evaluate cortical oscillations in a lesional-model, the human model system is a strength and weakness of the study. While experiments were not performed on overtly abnormal tissue, the neurological diseases affecting each subject may have altered their neurophysiology. Nonetheless, despite the chronic effects of disorders such as seizures and antiepileptic medications on the human cortex, cortical oscillations, and amplitude modulation are observed in epilepsy patients with invasive monitoring (Pfurtscheller and Berghold, 1989; Crone et al., 1998a,b). Thus, the results from such subjects still allow for interpretation of cortical oscillations and neuronal physiology. The recording location was anatomically distinct from the diseased area of brain by radiological examination in all patients. The recording site was 2.85 ± 0.59 cm away from pathology (Supplementary Table 1). In 10/10 subjects the cortical gray matter was normal by radiological examination. In 9/10 subjects, the entire neocortex around the recording site was normal by radiological examination (Figure 2; Supplementary Table 1). Histological examinations of recording area tissue were normal in 7/7 specimens (Figure 2; Supplementary Table 1). This normal cortex was resected to allow for removal of the patient's deeper target abnormal tissue; it was this normal region that was utilized for this study.

### Equipment

ECoG signals were recorded and digitized from the implanted electrode array at a sampling rate of 2400 Hz, using g.tech biosignal amplifiers. The unfiltered data were stored on a Dell PC running BCI2000 software. The ECoG electrodes (PMT® Corporation) were used to acquire ECoG signals. For two subjects 64 platinum electrodes were used. Four-contact electrodes were used for the remainder of experiments. Electrodes were made of platinum, each 4 mm in diameter with 2.3 mm exposed to the cortical surface and spaced apart by 1 cm. Recordings were made between electrodes along the same gyrus.

### Craniotomy

Each subject underwent a standard craniotomy under general sevoflurane anesthesia. Standard neurosurgical and neuroanesthesia practices were maintained to ensure similar craniotomy conditions. After removal of the cranial window and dural incision, the selected gyrus was exposed, ECoG electrodes were placed on the selected gyrus (Figure 1A) and location was confirmed with intraoperative MRI neuro-navigation (Figure 1B). An additional 4-contact distant electrode was placed over the inferior frontal gyrus perpendicular to the Sylvain fissure. For two subjects, a 64-channel electrode replaced the two separate 4-contract electrodes.

### Surgical transections and data collection

#### Intraoperative *in vivo* ECoG

Intraoperative *in vivo* ECoG data were collected from cortical tissue under general anesthesia. Two distant cortical electrodes were selected as ground and reference contacts and subject's left lower extremity was grounded. All surgical manipulation and operating room activity were halted during recordings. Prior to any recording, exquisite care was taken to ensure excellent contact between the pia and electrode surfaces. Adequate signal voltage recordings and signal-to-noise ratios were confirmed by an online BCI2000 graphics user interface. For each recording, 6 min epochs were collected with minimal operating room activity.

#### Surgical transections

Human ECoG was recorded before and after white and gray matter disruptions. Selective white matter transections eliminated white matter inputs underneath cortical tissue while maintaining surrounding gray matter inputs. Meanwhile, gray matter transections eliminated superficial gray matter inputs/outputs to the cortex of interest while preserving white matter connections. Experiments were only performed on tissues that were to be resected from a clinical standpoint and experimental plans had no bearing on clinical plans and decisions. Pre-procedural magnetic resonance imaging was evaluated for recording site tissue quality, distance to pathology and thickness of cortex. All transections were performed by a board-certified neurological surgeon subspecialized in epilepsy and brain-tumor surgery. Prior to experiments reported here, surgical transections were optimized and perfected to ensure to consistent lesions. All transections were directly visualized with expanded-field surgical telescopes (3.5X magnification) and complete transections were confirmed following each experiment. Most subjects received both white and gray matter disruptions in a sequential order. For some subjects, operative plan allowed only for use of one electrode. Three selective lesions were performed: (1) white matter transection, (2) gray matter transection or (3) combined gray/white transections (i.e., functional disconnection). White matter transection selectively and completely disrupted the gray-white junction under the electrode of interest without disrupting white matter under adjacent cortex and without disrupting surrounding gray matter. The precision of selective transections were confirmed post-hoc after experimental procedures. After baseline recording, a needle was used to puncture the pia matter adjacent to electrode #2 at four corners (1–4) surrounding the electrode (Figure 1C, *top*; Supplementary Figure 1). This puncture was created outside the recording strip. A straight sharp needle was bent at the tip to create a 90° bend with ~1 cm end. The angled needle was inserted into the puncture site and the bent portion was placed in the gray-white junction. Gray-white junction depth was determined by evaluating gyral architecture and gray matter thickness on preoperative MRI. The custom instrument was inserted in a fashion that would prevent gray matter tissue disruption (Figure 1C, Supplementary Figure 1). The bent portion of the instrument was then swept 90° toward the strip to selectively and completely disrupt the gray-white junction under electrode #2 without disrupting the gray matter. The instrument was then removed in a similar fashion as it was inserted to minimize tissue trauma and the procedure was repeated for the remaining three pin holes. Post-white matter lesion recording (“white”) was then performed.

Gray matter disruption selectively and completely disrupted the gray matter at a margin surrounding the electrode of interest without affecting the gray-white junction directly underneath. Gray matter lesions were performed in the adjacent electrode (Figure 1C, *bottom*). Four pinholes were created in the pia surrounding electrode #3. A blunt needle was bent at the tip to allow for subpial vertical transections similarly as described (Morrell et al., 1989). The probe was introduced through the pinhole opening next to electrode #3. The handle was gently raised up to isolate the cortical tissue of electrode #3 from adjacent gray matter. Care was taken to avoid disrupting the gray matter or gray-white junction directly underneath electrode #3. For vertical subpial transections, the edge of the instrument was gently raised against the pia without penetration. Vertical subpial transections were performed in 4 planes surrounding the electrode through each of the pial holes (1 to 4). Finally, combination of the white matter lesion at the underlying gray-white junction and gray matter transection of surrounding gray matter functionally isolated the cortex while maintaining pial blood supply. The complete functional disconnections of cortex was performed by disrupting surround gray matter around electrode #2 (which previously had a white matter disruption). For some subjects, a single electrode was used allowing for only a single transection experiment followed by functional disconnection. During the procedure, the pia and pial blood supply were maintained intact. Technical precision allowed for preservation of cortical vessels and bleeding was controlled by application of a thrombin-soaked gelatin sponge. Sham controls included electrodes over nearby non-transected cortex. Sham data included ECoG at baseline and after experiments at distant electrodes.

After procedures, complete transections were confirmed by visual inspection of all specimens through expanded-field surgical telescopes (3.5X magnification) which can identify white and gray matter similarly to traditional gross pathology specimens (Figure 2A). Accurate, precise and complete transections were confirmed in all specimens by gross specimen examinations. For 7 subjects, transections were performed in 2 electrodes, such that in electrode 2, white matter transections preceded complete disconnection and in electrode 3, gray matter transections preceded complete disconnection. For 3 subjects, on a single electrode was used and for these, a white matter transection preceded complete transection. There were no qualitative differences noted depending on which order the lesions were performed: a complete transection (i.e., “both”) produced similar results no matter which order the transections had been performed. For select samples, functionally-disconnected cortex was explanted, fixed in 10% formalin, stained in hematoxylin and eosin and examined by a neuropathologist. Exemplar microscopy of transected gray and white matter edges are demonstrated in Figures 2B,C.

#### Explant ECoG

Immediately after white and gray matter transections, the pia was carefully incised and the disconnected cortex was placed into a custom chamber with room-temperature Lactated Ringer's solution (130 mM Sodium, 109 mM Chloride, 28 mM lactate, 4 mM potassium, 2.7 mM Calcium). A 4-contact electrode was placed over the correctly-oriented cortex with the cortex under electrode #2 and the remaining electrodes in buffer. Electrodes 3 and 4 served as reference and ground, respectively. ECoG of freshly explanted tissue was recorded within 2 min of explanation. Operating room noise was measured by placing two 4-contract electrode strips into room temperature lactated ringers solution. After designating two of the eight electrodes and reference and ground, ECoG noise data were collected with equivalent operating room equipment and activity.

### Biomedical signal processing

All signal processing scripts were custom written in MATLAB, unless otherwise noted. Signals from every electrode were visually inspected and those electrodes identified as having predominately poor signal-to-noise characteristics (amplitude greater than 10x that of the majority of electrodes in the array) were excluded from further analysis. Less than 5% of data acquired were excluded due to poor signal-to-noise characteristics. Data were also excluded if there were incomplete transections (on gross tissue evaluation), intraoperative inconsistencies or incomplete data. For *in vivo* recordings, the signal at each remaining electrode were rereferenced to a distant frontal cortical electrode to minimize common sources of noise from the signals. *Ex vivo* explant data was re-referenced to the remaining adjacent electrode. Operating room noise measurements were referenced similarly. All re-referenced ECoG voltages were then processed through digital 0.5 Hz high-pass and 60-Hz-notch band-stop digital butterworth-filters.

#### Spectral power analysis

Spectral analysis was done using the Welch's power spectrum density estimate method (Welch, 1967). Welch's power spectrum density estimate divides the time series data into overlapping segments, computing a modified periodogram of each segment, and then averaging the power spectral density estimates. Spectral smoothing was performed using the Welch's power spectrum density estimate function in Matlab which calculated spectra on short segments with a windowing function. Hamming window was 10 times the sampling frequency with an overlap of 50% of each window and desired frequency resolution was 1 Hz. After power spectral densities of rereferenced signal voltages were calculated, the change in the logarithms of power spectral densities from baseline were then calculated and plotted. Traditional frequency bands were compared by binning data by frequency into δ (0.5–4 Hz), θ (4–7 Hz), α (8–12 Hz), β (12–30 Hz), γ (30–100 Hz), and high-γ (70–90 Hz). Measurements were compared to sham control which received no lesion.

#### Mutual information analysis

**Figure 3:**
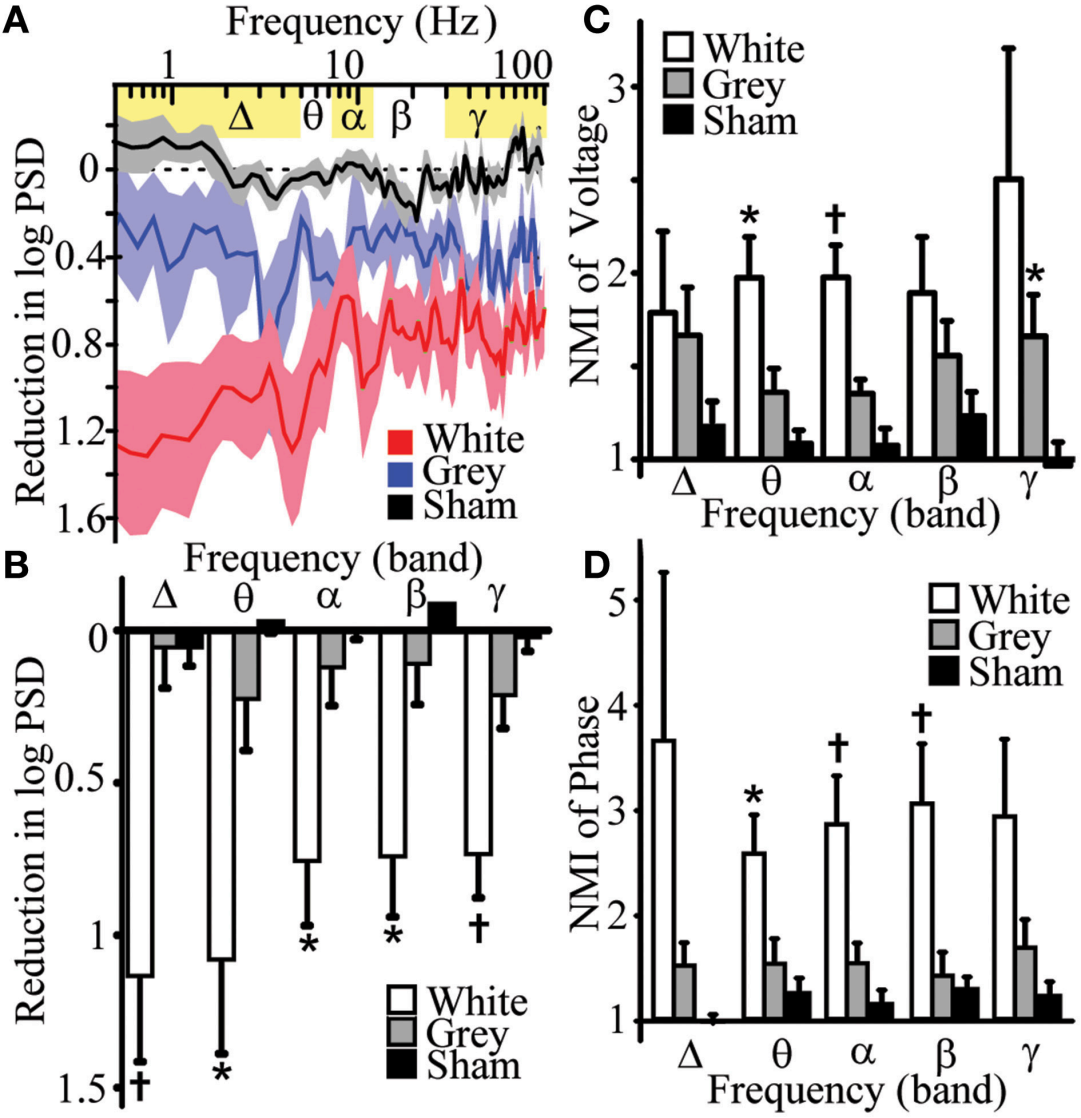
**Differential effects of white and gray matter transections on human cortical oscillation power spectra and mutual information. (A)** Reduction of logarithmic power spectral density (PSD) after white and gray matter disruptions vs. sham control. White matter transection affected frequency-dependent PSD (*p* = 0.001). **(B)** Reductions in PSD of band-specific frequencies after white (*n* = 7) and gray matter disruptions (*n* = 6) vs. sham control (*n* = 7). **(C)** Normalized mutual information (NMI, mutual information_post-*transection*_/mutual information_baseline_) of voltage with adjacent electrode after white and gray matter disruption vs. sham controls. **(D)** NMI of phase with adjacent electrode after white and gray matter disruption vs. sham controls (*n* = 9–11). Data represent mean ± s.e.m.; ^*^p < 0.05, vs. sham control. ^†^*p* < 0.05, vs. sham and gray matter disruption.

Mutual information is a quantification of the information gained about a random variable X from measurement of a second variable Y (Cover and Thomas, 1991; Leuthardt et al., 2009). Mutual information can measure non-monotonic and other more complicated relationships when compared to conventional measures such as correlation. Mutual information was used to assess functional connectivity between adjacent electrodes before and after select lesions. The MI is zero if X and Y are completely independent variables while the MI between X and Y will be >0 if knowing Y reduces the uncertainty of X. Information from measurements are represented as entropy (H), which can be used to calculate the MI between X and Y:

$$\begin{array}{l} \text{ H}\left(\text{X}\right)=\int_{-\infty }^{\infty }{\text{P}}_{\text{X}}\left(\text{X}\right)\;\log2\left({\text{P}}_{\text{X}}\left(\text{X}\right)\right)\\ \text{MI}(\text{X},\text{Y})\;=\text{ H}(\text{X})\;+\text{ H}(\text{Y})-\text{ H}(\text{X}|\text{Y}) \end{array}$$

where X is a voltage or phase time series, P_x_(x) is the marginal probability density of time series X or probability that X = x in system X, H(X) is the marginal entropy of system X, and H(X|Y) is the information of X gained by measurement of Y. To assess effects of lesions on functional connectivity, mutual information of band-passed signal voltage magnitudes (Figure 3C) and phase (Figure 3D) were measured between adjacent electrodes. The phase was determined by calculating by phase angle of the voltage signal magnitude in Hilbert space. To quantify information sharing after a specific lesion and control of inter-subject variability, mutual information was then re-referenced to baseline condition,

$$\begin{array}{l} \text{Normalized Mutual Information }(\text{NMI})\;=\\ \;\frac{{\text{Mutual Information}}_{\text{Post-transection}}}{{\text{Mutual Information}}_{\text{Baseline}}} \end{array}$$

and then compared to sham. Effects on mutual information were assessed by measuring the number of experiments showing a directional change relative to chance (via binomial probability) and comparing magnitudes of change. A directional general change was considered significant when the number of experiments showing specific changes in mutual information exceeded the number expected by chance (binomial test success probability<0.05).

#### Phase–amplitude coupling analysis

Phase-amplitude coupling signal analyses was performed to study how the phases of low frequency oscillations modulate the amplitude of high frequency oscillations within a single region of cortex, similarly as previously described (Canolty et al., 2006; Tort et al., 2010; Daitch et al., 2013). Phase-amplitude coupling for high-γ was assessed for every frequency pair in a 2-dimentional frequency space. PAC was performed at the electrode around/under-which transections were completed. One-Hz-width frequency bins from 1 to 25 Hz were used for phase and 1-Hz-width frequency bins from 70 to 90 Hz were used for amplitude. Spectral decomposition of the rereferenced signals was performed using Gabor wavelet filtering to produce instantaneous amplitude and phase estimates at time points for each frequency and then tailor the temporal resolution for each frequency (Canolty et al., 2006). Frequency-specific standard deviations of Gaussian envelopes were calculated around central frequencies. This allowed for sufficient full width at half-maximum bandwidth necessary for phase-amplitude coupling measurements through the beta frequency band. Magnitude of phase-amplitude coupling was represented as a modulation index Z-score, which represents the dependence of amplitude of one variable on the phase of another variable. Modulation index was calculated by applying an entropy measurement to determine divergence of the observed amplitude distribution from the uniform distribution (Tort et al., 2010). Data are shown as modulation index Z-score or change in modulation index Z-score from baseline (Canolty et al., 2006):

$$\text{z}(\text{t})=\text{A}(\text{t}){\text{e}}^{\left(\text{i})(\text{t}\right)}$$

where z(t) is the individual raw phase amplitude modulation time series, A(t) is the analytic amplitude time series (i.e., the amplitude envelope), and ϕ(t) is the phase time series.

$${\text{M}}_{\text{NORM}}=({\text{M}}_{\text{RAW}}-\mu )\text{/}\sigma$$

Where M_NORM_ is the normalized modulation index Z-score, M_RAW_ is the mean of z(t), μ is mean of surrogate lengths and σ is their standard deviation.

After correcting for multiple comparisons, a modulation index Z-score of 3.9 or greater indicated significant phase-amplitude coupling (*p* = 0.0494, *Z* = 3.9). The low frequency phase at phase amplitude coupling represents the mean phase at every instantaneous amplitude-phase complex. A phase of zero represented a peak. Each mean phase was displayed on a rose plot and distributions of mean phases were plotted histograms to show “preferred” phase, or phase that is tied to maximum amplitude of high-frequency power. Kurtosis (Pewsey, 2004; Berens, 2009) and concentration parameters of a von Mises distribution, κ (Berens, 2009), were calculated across all phase data points for each condition using the CircStat Matlab Toolbox(Berens, 2009) in order to quantitate phase distribution. To measure peakedness of the preferred phase distribution, circular kurtosis (Pewsey, 2004; Berens, 2009) was computed as

$$k=\frac{1}{N}\sum_{i=1}^{N}\cos2\left(\alpha_{i}-\overset{-}{\alpha }\right)$$

Where α was the angle is the angular direction/phase. In this measure, the larger the value toward 1 indicates a strongly peaked distribution.

### Statistics

To assess for statistical differences between multiple variables, multi-way analysis of variance tests (ANOVA) were performed on balanced groups. Data from 7 of the subjects (1, 3–5, 7, 8, 10) allowed for accurate comparisons for balanced n-way ANOVA statistics. Two-factor interactions were calculated due to limited factor combinations. In order to control for inter-subject and inter-electrode variability, data were normalized to pre-lesion data. When significant effects were measured, planned *post-hoc* Tukey's honestly significant differences were performed on balanced groups. All significant differences found on *post-hoc* comparisons had *p* < 0.05. Frequency-band data for lesion vs. sham were compared using planned unpaired Student's *t*-test across participants with Šidák multiple comparisons corrections (Sidak, 1967):

$$\alpha =1-{\left(1-\alpha \right)}^{1/\text{m}}$$

where m is the number of frequency band comparisons.

Binomial probability of changes in power and mutual information vs. chance were calculated. The binomial probability of observing k successes in N trials was:

$$\sum_{k=s}^{S2}\left(\begin{array}{c} N\\ k \end{array}\right)p^{k}{\left(1-p\right)}^{N-k}$$

Differences in preferred phase of PAC were assessed visually by plotting distribution of phase before and after transections on histograms and assessed statistically by comparing concentration parameters, κ, with circular statistics κ-test, which employs Bartlett's test (Snedecor and Cochran, 1989; Berens, 2009). Visual inspection of dataset histograms from each subjects and Jarque-Bera tests of normality were performed. *P*-values, after multiple comparison correction, degrees of freedom and F- and T-statistics are reported in the manuscript or supporting materials. Statistical values with *p* < 0.05 indicated statistical significance.

## Results

### Histological and radiographic evaluation of recording sites

Accurate transections of gray and white matter were performed with a custom instrument and delicate subpial technique. This ensured that pial blood supply was maintained during each experiment and complete transections were performed. Correct and precise selective gray and white matter transections were confirmed during each experiment by visual inspection of gross specimens through expanded-field surgical telescopes (3.5X magnification) which can identify white and gray matter and also by detailed histological examination (Figures 2A–C).

Despite the substantial utility of *in vivo* human cortical transections, it was imperative to confirm that the experimental tissue did not show abnormalities and were not affected by the pathology that the surgery was intended to address (e.g., a seizure focus). To reduce the impact of disease on experimental results, candidates were only selected for this study if the site of each experimental recording would be performed a significant distance away from the pathological region. All of the operations required the unavoidable surgical resection of normal brain tissue in order to access the pathological region. Thus, the region of abnormality (i.e., epileptogenic zone) was quite distant from the region tested in these experiments. Despite the notable distance from pathology, detailed radiological, and histological examinations of recording sites were performed. These experiments demonstrated that recordings were not performed on abnormal tissue. First, high-resolution magnetic resonance imaging of the brain was performed on each subject and T1-, T2-, and diffusion-weighted sequences were directly evaluated by a neuroradiologist for causes and consequences of epilepsy and other pathology. Radiographic examination of the recorded gray matter tissue showed no abnormal tissue in 10/10 subjects (Figures 2D,E, Supplementary Table 1). Second, detailed neuropathological histological examinations failed to show any abnormalities in lamination, architecture, cell morphology or parenchymal changes (i.e., no histological causes or consequences of epilepsy or other pathology) in 7/7 recording samples (Figures 2F,J, Supplementary Table 1). Hence, experiments were performed on cortex with neither radiographic nor histological abnormalities and cortex which was distant from the region of pathology.

### White matter circuits contribute to power of low and high frequency oscillations

Historically, lower frequency rhythms are thought to represent longer range circuits such as thalamocortical interactions (Pfurtscheller, 1981; Crone et al., 1998b; Miller et al., 2007), whereas local cortical circuits and action potential firing have been associated with higher frequency gamma rhythms (Crone et al., 1998a; Manning et al., 2009). We anticipated that white and gray matter manipulations would preferentially impact low and high frequencies, respectively. To evaluate the contributions of white and gray matter connections to local power of human cortical oscillations, power spectral density (PSD, Supplementary Figure 2) estimates were measured following selective transections. Three distinct statistical methods were used to assess for lesion-effects on PSD. Multi-way ANOVA showed that white matter disruption significantly reduced frequency-dependent logarithmic PSD (frequency-condition interaction) when compared to sham control [Figures 3A,B, Supplementary Table 2, *top*; analysis of variance, ANOVA *p*_*a*_ = 0.001, *F*_(89, 534)_ = 1.59]. *Post hoc* Tukey's honestly significant difference analysis after ANOVA showed white matter lesions reduced PSD in 100% of δ-band frequencies but only 46% of γ-band frequencies tested. Analysis of band-passed power frequencies showed that white-matter disruption reduced PSD in all frequency bands (δ, θ, α, β, and γ; Student's *t*-test *p*_*t*_ < 0.05, Figures 3A,B, Supplementary Table 2, *bottom*). Finally, white matter transection reduced PSD in all subjects for δ, β, and γ frequency band (binomial probability, *p*_*bp*_ = 0.0078; Supplementary Table 2, *bottom*;). Each of these analyses showed that white matter transection reduced power in low and high frequency domains suggesting that white matter circuits contribute to power of cortical oscillations.

Although gray matter disruption had a main effect on PSD [Figure 3A, Supplementary Table 2, *top*; *p*_*a*_ < 0.001, *F*_(1, 445)_ = 58.6], it had no significant effect on frequency-dependent PSD [Figure 3A, Supplementary Table 2, *top*; *p*_*a*_ = 0.067, *F*_(89, 445)_ = 1.26] or on PSD of canonical frequency bands (Figure 3B, Supplementary Table 2, *bottom*). Hence, white matter transection reduced PSD in lower frequency power but also reduced gamma power as well. However, transections of surrounding gray matter had no significant effects on the power of both low- and high-frequency oscillations. This can be interpreted to suggest that gray matter circuits between cortex does not contribute to the power of local oscillations

### White and gray matter circuits maintain topographic heterogeneity and autonomy

Functional connectivity measures are statistical methods that can be used to estimate interactions between neighboring regions of cortex and have been applied to electrophysiology and functional neuroimaging studies (Cover and Thomas, 1991; Ortega et al., 2008; Friston, 2011; Greenblatt et al., 2012). To assess functional connectivity, mutual information shared with adjacent regions of cortex was measured after selective disruption of white and gray matter. The hypotheses predicted reduced mutual information for lower frequencies after white matter transections and reduced mutual information for higher frequency after the gray matter transections. Contrary to our expectation, white and gray matter disruptions differentially increased functional connectivity at select frequency bands.

Normalized mutual information (NMI) of voltage amplitude and phase were assessed between electrodes before and after white or gray matter transections. NMI of voltage amplitudes assessed global information shared between electrodes. Meanwhile, NMI of phase assessed amplitude-independent relationships fundamental in advanced signal processing such as phase-amplitude coupling.

NMI of voltage amplitudes were increased after white matter disruptions in adjacent cortex for θ, α, β, and γ bands for a significant proportion of experiments (binomial probability of reduction, *p*_*bp*_ < 0.05; Supplementary Table 3). *Magnitude* of NMI for voltage amplitude increased in θ and α bands after white matter disruption (Figure 3C, Supplementary Table 3; *p*_*t*_ < 0.05). This suggests that white matter disruption generally increases global shared information, but did so more robustly in lower frequency domains. To compare amplitude-*independent* relationships of cortical oscillations, NMI of oscillation phases were measured. White matter disruptions increased NMI of phase for all frequency bands in a significant proportion of experiments (*p*_*bp*_ < 0.03; Supplementary Table 4). Magnitude of phase-based NMI increased in θ, α, and β bands after white matter disruption (Figure 3D, Supplementary Table 4; *p*_*t*_ < 0.03). This finding suggests that white matter disruption increased information shared for amplitude-independent phase, particularly in low frequency domains. Together the results support the hypothesis that white matter connections facilitate spatial heterogeneity especially for low-frequency oscillations.

NMI of voltage amplitudes were increased after gray matter disruptions in adjacent cortex for θ, α, β, and γ bands for a significant proportion of experiments (binomial probability of reduction, *p*_*bp*_ < 0.05; Supplementary Table 3). Magnitude of NMI for voltage amplitude increased in γ band after gray matter disruption (Figure 3C, Supplementary Table 3; *p*_*t*_ < 0.05). Gray matter transection did increase NMI of phase for θ, α, and γ bands in significant proportion of experiments (*p*_*bp*_ < 0.05) but did not significantly affect magnitudes for NMI of phase in any band. White matter disruption increased NMI more than gray matter disruption for voltage and phase in α and α-β bands, respectively (*p*_*t*_ < 0.05). These finding suggest that gray matter disruption had global effects on shared information but meaningfully increased shared information magnitude of amplitude-dependent oscillations for high frequencies. This further insinuates that gray matter circuits play a limited but measurable role in managing spatial heterogeneity, especially for high-frequency oscillations. Hence, disruption of white and gray matter differentially *increased* mutual information shared with adjacent cortex.

### Gray matter circuits provide temporal segregation of nested oscillations

**Figure 4:**
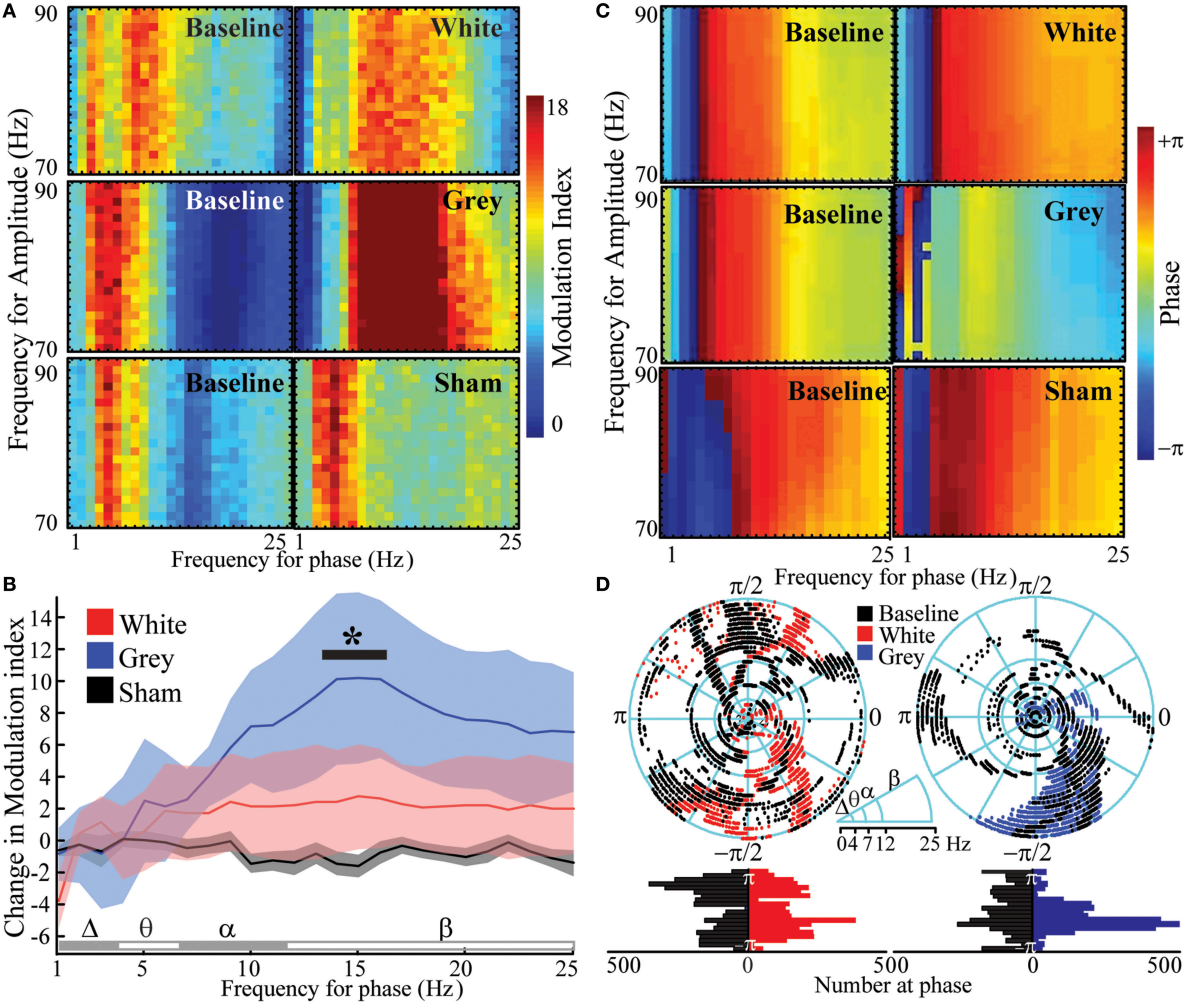
**Increased β- phase- γ amplitude coupling and synchronized phase of coupling after gray matter disruption. (A)** Cross-frequency phase-amplitude coupling with high-γ power (PAC) showing modulation indices at baseline and after white, gray or sham disruptions in an exemplar subject. **(B)** Mean change in PAC modulation indices from baseline for white, gray and sham disruptions from all subjects. **(C)** Phase at PAC at baseline and after white, gray or sham disruptions in exemplar subjects. **(D)** Rose plots and histograms showing preferred phase for PAC at baseline and after disruptions in all subjects. Note the transition from a diversity of PAC relationships to uniform phase entrainment after gray-matter disruption. *N* = 6 for white and gray lesions and *n* = 12 for shams. Gray matter disruption significantly affected frequency-dependent PAC vs. sham (*p* = 0.002; ^*^p < 0.05, *post-hoc* Tukey's test). Data represent mean ± s.e.m.

Cross-frequency phase-amplitude coupling (PAC) between low-frequency phases and high-γ amplitude has been implicated in neuronal firing, cortical activation, task completion, and alterations in consciousness (Canolty et al., 2006; Cardin et al., 2009; Breshears et al., 2010). PAC has been suggested to serve as a mechanism to transfer information from large-scale distributed brain networks to the fast, local cortical processing required for effective computation and synaptic modification (Canolty and Knight, 2010). We hypothesized that if PAC was primarily a cortical-cortical form of communication, gray matter transections should reduce PAC within a region or cortex; if this phenomenon was centrally mediated, on the other hand, white matter transection should reduce this nesting rhythm structure. The results again ran counter to our expectations. Analyses of variances were performed to assess for changes in PAC after each lesion. Gray matter disruption [*p*_*a*_ = 0.002, *F*_(24, 120)_ = 2.26] but not white matter disruption [*p*_*a*_ = 0.334, *F*_(24, 120)_ = 1.12] significantly affected frequency-dependent PAC (frequency-condition interaction; Figures 4A,B, Supplementary Table 5). *Post hoc* comparisons showed low β-phase to γ amplitude PAC was increased after gray matter disruption vs. sham. The preferred phase for maximal PAC was broadly distributed at baseline [Figures 4C,D; κ(concentration parameter) = 0.37; circular kurtosis = 0.14] and after white matter disruption [κ = 0.33; kurtosis = 0.03; κ-test, *p*_κ_ = 0.54, *F*_(6298)_ = 1.02]. However, rose plots demonstrated that gray matter disruption synchronized phase for PAC, especially for β-γ resulting in a more strongly peaked distribution after gray matter disruption (κ = 1.70; kurtosis = 0.30) vs. baseline [κ = 0.27; kurtosis = 0.10; *p*_κ_ = 0, *F*(6298) = 2.42; Figures 4C,D]. Hence, gray matter disruption increased β-γ PAC and synchronized preferred phase for PAC. Sham lesion (κ = 0.93; kurtosis = 0.32) did not affect distribution of preferred phase for PAC relative to baseline [κ = 0.92; kurtosis = 0.38; *p*_κ_ = 0.86, *F*_(6298)_ = 1.01; Supplementary Figure 3]. Where white matter transection previously showed a reduction in the heterogeneity of cortical-cortical interactions (i.e., increase in NMI), gray matter transection reveals a reduction in the heterogeneity of phase entrainment (i.e., an increase in PAC at a preferred phase). Thus, where white matter-mediated circuits seems to play a larger role in spatial segregation of oscillatory behavior, gray matter connections play a larger role in its temporal segregation of nested oscillations.

### Human cortex consists of independent oscillatory units

**Figure 5:**
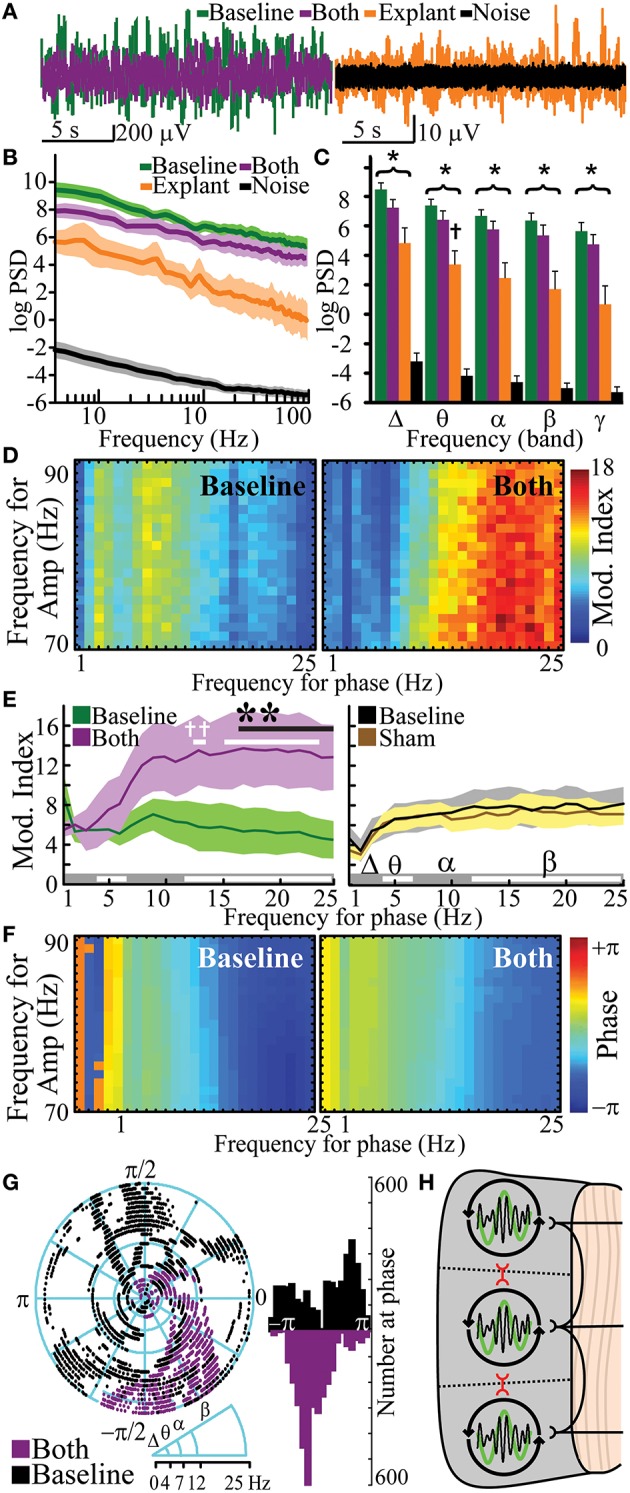
**Human cortical oscillations from functionally disconnected cortical gray matter**. Exemplar recordings of cortical oscillations **(A)**, logarithmic PSD **(B)**, and frequency band-specific PSD **(C)** of baseline (*n* = 7), combined gray/white matter disruption (*both*, i.e., functionally-disconnection crotex, *n* = 7) and tissue explant (*n* = 5) vs. operating room noise (*n* = 36). **(D)** Representative cross-frequency PAC for high-γ amplitude showing modulation indices (Mod. Index) at baseline and after combined gray/white disruption. **(E)** Quantitation of modulation index baseline and after combined disruption (*n* = 6) vs. sham control (*n* = 12). **(F,G)** Representative phase at PAC at baseline and after combined disruptions with quantitation and distribution analysis. **(H)** Model for neural networks responsible for cortical oscillations, in which white matter fibers facilitate independent oscillatory units (IOUs) within gray matter while contributing to heterogeneity between IOUs. Gray matter circuits between IOUs (red) also facilitate topographical heterogeneity by attenuating PAC. ^*^p < 0.05, vs. noise PSD; ^†^*p* < 0.05, vs. baseline PSD. Functional disconnection significantly changed frequency-dependent PAC vs. baseline (*p* < 10^−4^); ^**^p < 0.05, *post-hoc*, vs. baseline, ^†^*p* < 0.05, *post-hoc*, vs. 1 Hz. Data represent mean ± s.e.m.

In addition to defining the influence that short range (gray matter) and long range (white matter) connections had on the oscillatory behavior of cortex by their independent transection, we further characterized true local rhythms by combining these transections to see what rhythmic behavior remained after a full functional disconnection. We hypothesized that cortex, devoid of inputs, was still capable of generating cortical oscillations. Functional disconnection of cortex had a significant negative effect on PSD [Figures 5A,B; *p*_*a*_ = 3.7 × 10^−213^, *F*(1, 534) = 2761] and reduced broad-band PSD in all experiments (7/7 experiments, *p*_*bp*_ = 0.0078). Despite disruption of all input and output circuits, functionally-disconnected cortex had significantly more frequency-dependent cortical oscillations than experimental noise [frequency-condition interaction; Figures 5A,B; *p*_*a*_ = 1.9 × 10^−80^, *F*_(89, 534)_ = 11.69]. In *post-hoc* analysis, PSDs of functionally disconnected cortex were significantly different from noise at all frequencies between 1 and 100 Hz. When binned into canonical frequency bands, functionally-disconnected cortex also had cortical oscillation PSD greater than noise in all bands (Figure 5C, Supplementary Table 6; *p*_*t*_ < 10^−5^). To further control for the potential effect of volume conduction from adjacent connected tissue, ECoG measurements were then recorded from extracted cortical brain tissue. Physical explant (and devascularization) led to a frequency-dependent reduction in power when compared to functional disconnection [frequency-condition interaction; *p*_*a*_ = 1.7 × 10^−39^, *F*_(89, 356)_ = 6.77]. *Post hoc* analysis showed all frequencies had population means significantly different from functional disconnection. When binned into frequency bands, explant's PSD was different from baseline in the θ band (*p*_*t*_ = 0.04). Despite physical extraction, cortical oscillations from explanted cortex had significantly greater frequency-dependent PSD than noise [frequency-condition interaction; *p*_*a*_ = 1.6 × 10^−34^, *F*_(89, 356)_ = 5.98] and, in *post-hoc* comparisons, PSD was greater than noise for β-θ frequencies. Explant's PSD was also greater than noise in all frequencies when binned into canonical frequency bands (Figure 5C, Supplementary Table 6; *p*_*t*_ < 0.05). Functional isolation of the cortex led to highly variable changes in functional connectivity resulting in insignificant shared information (Supplementary Figure 4). The oscillations seen in explanted cortex suggest an intrinsic component rather than pure volume conduction. Hence, functionally disconnected and physically explanted cortex exhibits persistent cortical oscillations.

### Functionally isolated IOUs lose temporal diversity in PAC

Pharmacologically-induced cortical PAC has been observed in slice models *in vitro* (Florez et al., 2013). However, PAC has not been observed in intact but functionally disconnected cortex. PAC was evaluated after functionally isolating cortex. Functional disconnection of cortex led to a frequency-dependent increase in PAC (frequency-condition interaction) when compared to baseline [Figures 5D,E, Supplementary Table 7; *p*_*a*_ = 7.4 × 10^−5^, *F*_(24, 120)_ = 2.90] and when compared to sham [*p*_*a*_ = 5.31 × 10^−5^, *F*_(24, 120)_ = 2.96]. *Post hoc* comparisons showed that PAC was greater than baseline for β frequencies (17–25 Hz). Furthermore, *post-hoc* analysis showed that β frequencies in functionally disconnected cortex had significantly greater PAC than the 1 Hz δ frequency. Sham had no significant effect on frequency-dependent PAC [*p*_*a*_ = 0.56, *F*_(24, 120)_ = 0.93]. Similar to the gray matter disruption, complete functional disconnection of cortex synchronized phase of PAC [Figures 5F,G; κ = 1.78; kurtosis = 0.32; *p*_κ_ = 0 vs. baseline, *F*(6298) = 2.40]. PAC was observed in 1/5 explanted specimens (Supplementary Figure 5). PAC would not be an expected finding in pure volume conduction, suggesting a phenomenon intrinsic to the disconnected cortex. Thus, functional disconnection of cortex enhanced β-γ PAC at a synchronized phase.

## Discussion

Selective lesions, functional disconnection and ultimately full explant demonstrate that mesoscale regions of cortex can independently generate complex oscillations and oscillatory relationships. Several previous studies support the existence of these IOUs. Amzica et al. observed cortical oscillations after disrupting deep circuits in anesthetized cats (Amzica and Steriade, 1995). Ponce et al. demonstrated independent phase oscillators in human cortex measured by functional magnetic resonance imaging (Ponce-Alvarez et al., 2015). There are numerous studies that demonstrate cortical oscillations in disconnected cortical tissue including cultures, explants, and *in vitro* slices (Kristiansen and Courtois, 1949; Mody et al., 1987; Chagnac-Amitai and Connors, 1989a,b; Maeda et al., 1995; Kohling et al., 1998; Whittington et al., 1998; Timofeev et al., 2000; Sohal et al., 2009; Florez et al., 2013). These IOUs are further differentially modulated by their relationships with other IOUs (i.e., gray matter connections) and by their relationships with deeper structures via their white matter connections (Figure 5H). When these IOUs are fully isolated, β-γ PAC is the default organizing mechanism. Some prior data support this hypothesis; for example, patients with dysfunctional cortical feed-back circuits, such as those with Parkinson's disease, demonstrate exaggerated β-γ PAC (De Hemptinne et al., 2013). This rhythm structure is primarily modulated by neighboring suppression from adjacent cortical-cortical connections. White matter connections, however, are associated primarily with facilitation of amplitude (for lower frequencies more than high). Independently perturbing these circuits leads to distinct types of reduction in the signal heterogeneity of these processing units. White matter lesions lead to more spatially homogeneous signals between cortical sites (i.e., an increase in NMI), while gray matter separation result in a more uniform phase temporal synchronization for PAC.

For white matter lesions, the bands that show the most notable increases in NMI are also similar to those with the most reduced power. This relationship could represent relatively increased input from the lesioned area's cortical neighbor through gray matter connections. Numerous prior studies support this finding that white matter connections facilitate spatial heterogeneity. Amzica et al. showed that disconnection of intracortical synaptic linkages in anesthetized cats disrupts synchronization of slow oscillations (Amzica and Steriade, 1995). Gupta et al. showed that long range connectivity as measured by functional magnetic resonance imaging was changed after diseases and this altered spatial heterogeneity (Gupta et al., 2014). Ibrahim et al. found that ipsilateral functional connectivity was increased after a functional hemispherotomy in which one hemisphere was disconnected from the other (Ibrahim et al., 2015).

It is also notable that when a cortical site's gray matter is circumferentially separated that there is an *increase* in shared information at the gamma band. The finding that information increases between separated regions at first seems counterintuitive, given that high-frequency rhythms are thought to represent local cortical circuits (Manning et al., 2009). We posit that this loss of cortical-cortical connectivity leads to a default oscillatory structure that is more similar rather than different from its cortical neighbor. Taken together, we interpret these finding to indicate that white matter and gray matter connections are important not only for shared cortical processing, but also for maintaining topographic heterogeneity and autonomy of oscillatory behavior in mesoscale regions of cortex.

The mid-twentieth-century was notable for a debate over cortical vs. subcortical origins for rhythmic electrical activity of the brain (Kristiansen and Courtois, 1949; Pedley and Traub, 1990). By the 1960s, the discussion of cortical oscillations was largely dominated by the theory of thalamic control (Kristiansen and Courtois, 1949; Andersen and Sears, 1964; Szerb, 1967; Andersen and Andersson, 1968; Ulmar et al., 1975; Pedley and Traub, 1990). Indeed, several other studies support the hypothesis that gray matter can independently produce cortical oscillations at a wide range of frequencies in absence of input from white matter (Llinas, 1988; Llinas et al., 1991, 1999; Timofeev and Steriade, 1996; Florez et al., 2013). When taken together, the data suggest that that local interneuron circuits play a key role in cortical-cortical signal processing (Pedley and Traub, 1990; Traub et al., 1998; Povysheva et al., 2008; Cardin et al., 2009; Sohal et al., 2009; Buzsaki and Wang, 2012) in conjunction with input and pacing from circuits traveling through white matter (Andersen and Sears, 1964; Buzaki, 2006; Bollimunta et al., 2008). Multiple groups have shown that thalamocortical circuits contribute to low frequency rhythms, most notably μ and β (Andersen and Sears, 1964; Andersen and Andersson, 1968; Pedley and Traub, 1990; Vertes and Kocsis, 1997; Engel et al., 2008; Buzsaki and Wang, 2012; Malekmohammadi et al., 2014). While low-frequency power is indeed reduced with a white matter transection, these low frequencies are still present when functionally and physically separated. Thus, this suggests low frequency oscillations are facilitated by white matter projections to maintain functional autonomy between adjacent IOUs but can be independently generated by gray matter cortical-cortical circuits. In addition to deep projections playing a role in functional heterogeneity of cortex, cortical-cortical interactions are also active in this process. We posit that independent cortical timing relies on the activity of inhibitory interneurons within the gray matter (Beggs and Plenz, 2003; Povysheva et al., 2008; Cardin et al., 2009; Sohal et al., 2009; Buzsaki and Wang, 2012; Jeschke and Ohl, 2012; Florez et al., 2013). In this model, gray matter afferents activate local inhibitory neurons that diminish PAC in adjacent cortex, thus maintaining a diversity of oscillatory relationships. When lost, there is a more generalized entrainment at a uniform phase.

There are several limitations and assumptions in this study that deserve attention. First, volume conduction presents problems for the interpretation of functional connectivity measures between signals recorded from adjacent regions of cortex (Buzsaki et al., 2012). Although some authors contend that volume conduction cannot explain high-frequency events measured by ECoG (Bahramisharif et al., 2013), it remains difficult to conclusively make distinctions between effects of synchrony due to volume conduction and synchrony as functional biological neural connectivity. Despite this omnipresent confound in neurophysiology, there are several lines of evidence to support that the findings presented here cannot be entirely attributed to volume conduction. After removing common, instantaneous, signal among electrodes, white matter transections reduced signal power, especially in low frequencies. This reduction in power cannot be explained by and is explicitly contrary to volume-conducted effects. Second, white matter disruption reduced power in δ-band frequencies more than higher frequencies but significantly increased the functional connectivity with adjacent cortex in θ, α, and β bands. Gray matter transections did not have a major effect on power but produced a selective increase in γ-band functional connectivity. These frequency band-specific findings changes in functional connectivity after transections are unlikely to be explained by persistent volume conduction. Third, increased β-γ PAC with gray matter transections would not be an expected finding with pure volume conduction artifact. Volume conduction would not enhance PAC and would maintain previous phase relationships; this supports a phenomenon pertaining to the disconnected circuits. Indeed, direct demonstration of a correlation between PAC and neuronal firing has been observed by multiple groups (Buzsaki et al., 2012). Finally, the oscillations seen in explanted cortex suggest an intrinsic component rather than pure volume conduction. When taken together, (1) the removal of instantaneous common signal, (2) frequency-specific reduction in power associated with white matter transection, (3) differential functional connectivity changes observed from selective transections, (4) enhancement of band-specific PAC after selective transection, (5) the known associations between PAC and neuronal firing, and (6) persistence of oscillations in explanted tissue attenuate concerns regarding volume conduction artifact.

It is well-known that the cytoarchitecture of cortex varies across the human brain (Amunts et al., 2007). While the results presented here were found in the temporal and frontal lobes, the degree to which our current findings can be generalized across other regions of the brain is not fully defined. Although the experiments were performed several centimeters away from pathology, pathology-specific effects may affect some of the findings. To maximize subject recruitment, data included subjects with epilepsy and brain tumors. Excluding subjects with brain tumors showed a trend toward similar results but the data were underpowered to demonstrate statistical significance. Combining the two subject populations revealed significant findings but underlying disease may affect interpretation. Future studies aimed at pathology specific affects are warranted. Some of the lesion experiments were performed in sequence, where the white matter lesions were performed after the gray matter lesions. Although, relative baselines were recalculated for each measurement, this adds an additional difficulty in interpretation. Many studies have studied high gamma band range up to and even beyond 150 Hz. This study was limited to gramma frequencies below 100 Hz due to noise within the operating room environment. Finally, many of the patients in this study had epilepsy and their general brain physiology may differ from that of the general population because of the presence of seizure foci and the chronic use of anti-epileptics. Despite this, the experiments were performed on radiographically and histologically normal tissue.

In conclusion, *in vivo* transections in the human brain demonstrates that mesoscale regions of cortex are independently capable of maintaining complex oscillatory behavior and the alteration of this activity and its cortical heterogeneity is differentially modulated by white and gray matter mediated circuits. This mechanism offers added insight into how the brain establishes functional diversity between adjacent cortical regions.

## Author contributions

AH, DB, and EL conceived and designed the experiments; AH and EL performed the experiments; AH, DK, NL, and SD analyzed the data; EL and DB supervised the research. All authors wrote the manuscript.

### Conflict of interest statement

The authors declare that the research was conducted in the absence of any commercial or financial relationships that could be construed as a potential conflict of interest. The reviewer KS and handling Editor declared their shared affiliation, and the handling Editor states that the process nevertheless met the standards of a fair and objective review.

## Supplementary material

Supplementary Figure 1 **Step-wise illustrations depicting subpial white matter disruption at the gray-white junction under electrode #2 (top) and selective gray matter disruptions around electrode #3 (bottom) are shown with contacts over middle temporal gyrus**. After creating four pinholes around electrode #2, a custom instrument was inserted into the gray-white junction without disrupting significant gray matter. The instrument was rotated 90° to disrupt the gray-white junction under electrode #2. This was repeated for each pin hole. For gray matter disruption, four pin holes were created around electrode #3. A custom instrument was inserted at the gray white junction though pinhole 1 and oriented between pinholes 1 and 2. The instrument was gently raised until reaching the pia matter to disrupt all gray matter between pinholes 1 and 2. The gray matter disruptions were repeated between each pinhole. The desired lesions are depicted in blue (*right*).

Supplementary Figure 2 **Intraoperative recordings of cortical oscillations**. Example cortical oscillations are shown (inset) with logarithmic PSD during baseline (*n* = 7). Dotted line highlight represents linear fit for >30 Hz oscillations and highlights peaks at alpha and delta at baseline.

Supplementary Figure 3 **Rose plot and histogram quantitation of cross-frequency PAC for high-γ amplitude showing similar phase distribution during baseline and after sham lesion (*n* = 11)**.

Supplementary Figure 4 **Effects of functional disconnection of cortex on shared information**. Normalized mutual information (NMI, mutual information_post−transection_/mutual information_baseline_) of voltage with adjacent electrode after combined white and gray matter disruption vs. sham controls (*n* = 10). Data represent mean ± s.e.m.

Supplementary Figure 5 **Cortical explant displays phase-amplitude coupling *ex vivo***. Cross-frequency phase-amplitude coupling for high-γ amplitude showing modulation indices **(A)** and preferred phase with quantitation **(B)**. Arrow indicates modulation index for *p* = 0.05.

Supplementary Table 1 **Demographic and clinical information**.

Supplementary Table 2 **Change in power spectral density: Statistics Data**.

Supplementary Table 3 **Normalized mutual information of voltage shared with adjacent cortex: Statistics data**.

Supplementary Table 4 **Normalized mutual information of phase shared with adjacent cortex: Statistics data**.

Supplementary Table 5 **Change in phase amplitude coupling of 1–25 Hz phases with 70–90 Hz amplitude: Statistics data**.

Supplementary Table 6 **Power spectral density after functional and physical disconnection from cortex**.

Supplementary Table 7 **phase amplitude coupling after functional disconnection: Statistics data**.

## Abbreviations

BP: Binomial Probability
ECoG: electrocorticography
ANOVA: multiway analysis of variance test
IOUs: independent oscillatory units
NMI: normalized mutual information
PSD: power spectral density estimate
PAC: phase-amplitude coupling. Band-specific δ, θ, α, μ, β, γ, and high-γ were defined as 0.5–4, 4–7, 7–12, 8–12, 12–30, 30–100, and 70–90 Hz, respectively.
