# Supplementary figures and images for: Influence of White and Gray Matter Connections on Endogenous Human Cortical Oscillations
Source: Front Hum Neurosci. 2016 Jun 28;10:330. doi: 10.3389/fnhum.2016.00330 (PMC4923146; doi:10.3389/fnhum.2016.00330)

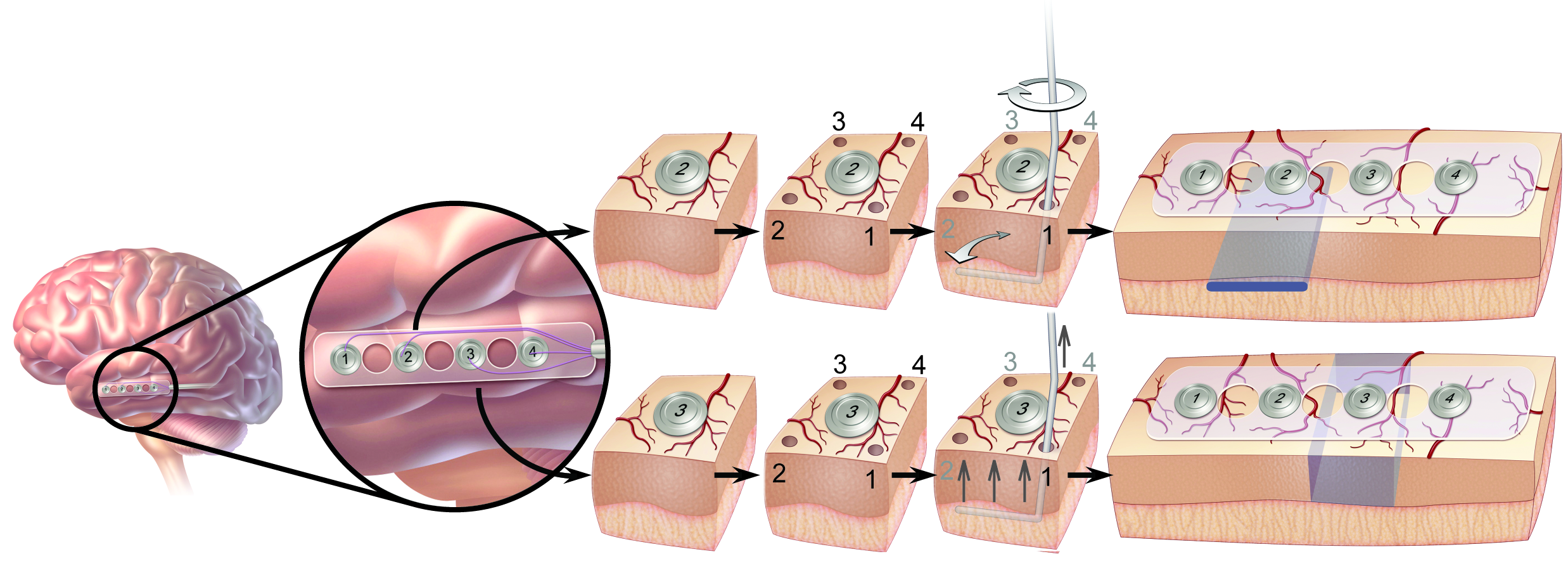

Supplement: Supplementary Figure 1 — Step-wise illustrations depicting subpial white matter disruption at the gray-white junction under electrode #2 (top) and selective gray matter disruptions around electrode #3 (bottom) are shown with contacts over middle temporal gyrus. After creating four pinholes around electrode #2, a custom instrument was inserted into the gray-white junction without disrupting significant gray matter. The instrument was rotated 90° to disrupt the gray-white junction under electrode #2. This was repeated for each pin hole. For gray matter disruption, four pin holes were created around electrode #3. A custom instrument was inserted at the gray white junction though pinhole 1 and oriented between pinholes 1 and 2. The instrument was gently raised until reaching the pia matter to disrupt all gray matter between pinholes 1 and 2. The gray matter disruptions were repeated between each pinhole. The desired lesions are depicted in blue (right). [file Image1.TIF]

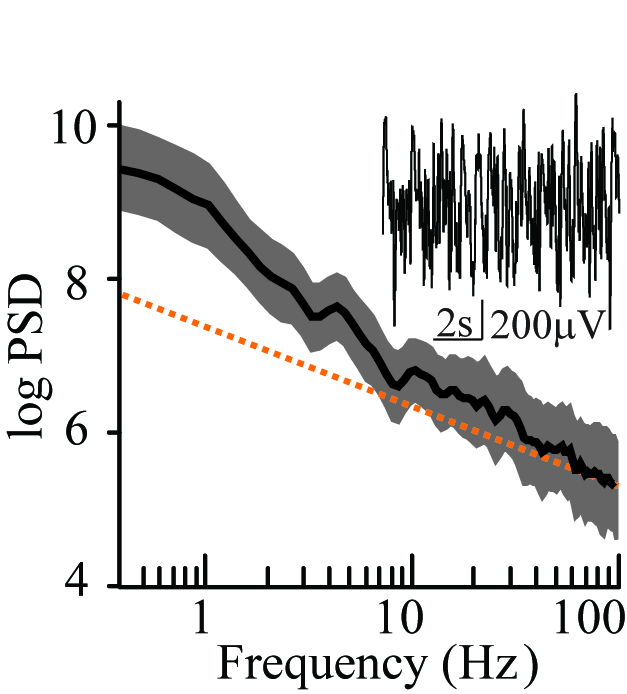

Supplement: Supplementary Figure 2 — Intraoperative recordings of cortical oscillations. Example cortical oscillations are shown (inset) with logarithmic PSD during baseline (n = 7). Dotted line highlight represents linear fit for >30 Hz oscillations and highlights peaks at alpha and delta at baseline. [file Image2.TIF]

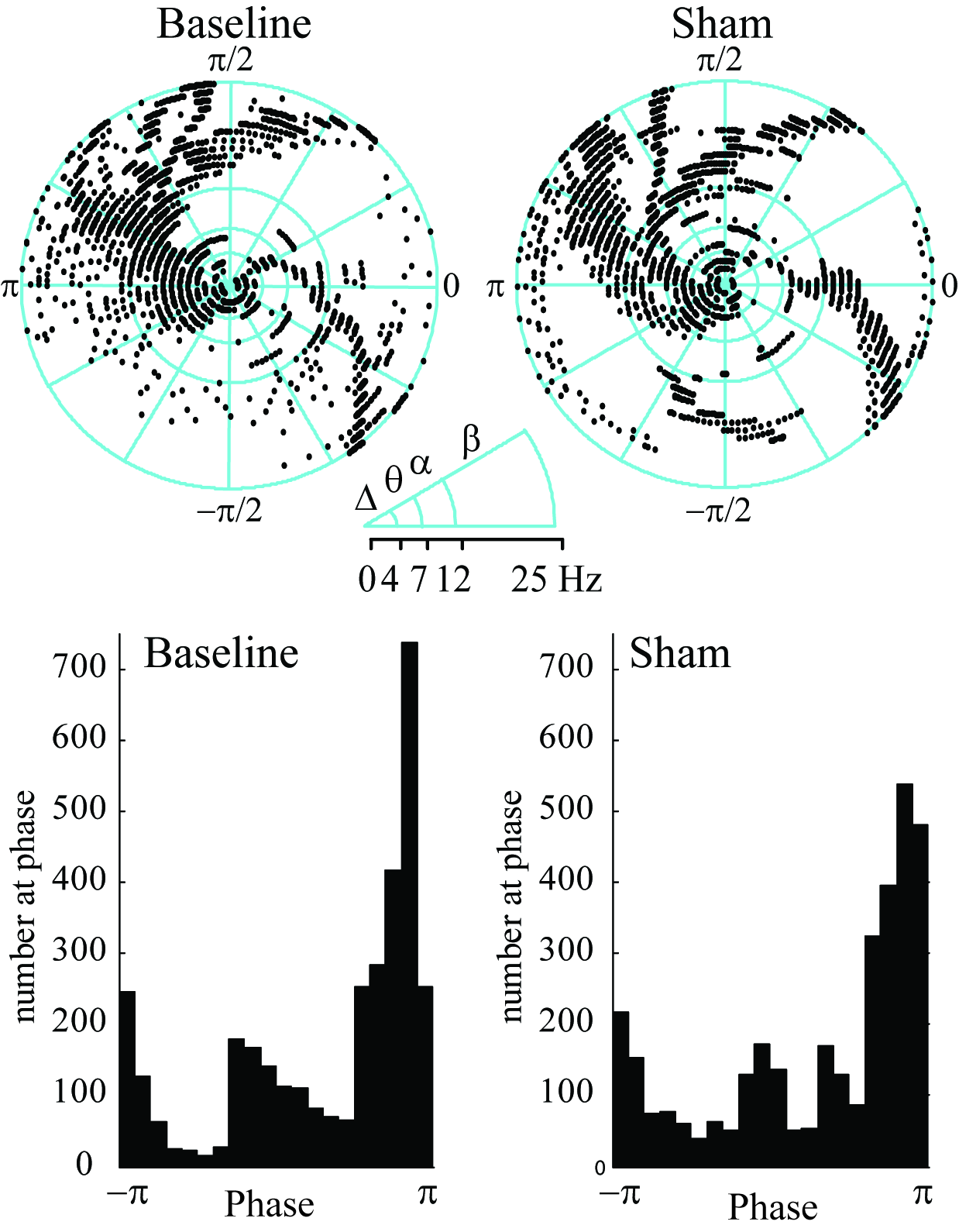

Supplement: Supplementary Figure 3 — Rose plot and histogram quantitation of cross-frequency PAC for high-γ amplitude showing similar phase distribution during baseline and after sham lesion (n = 11). [file Image3.TIF]

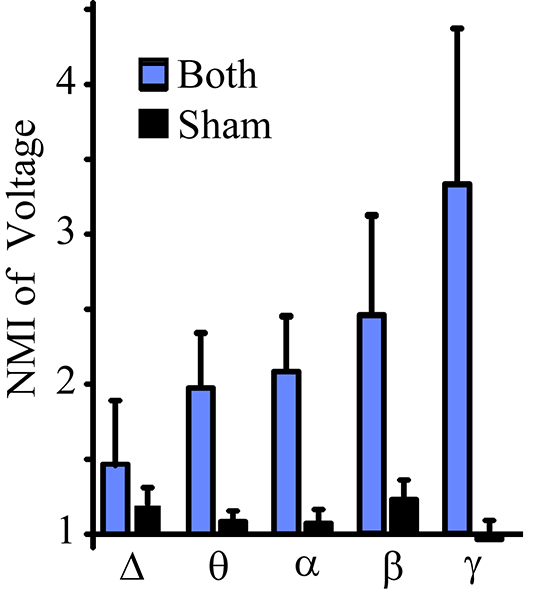

Supplement: Supplementary Figure 4 — Effects of functional disconnection of cortex on shared information. Normalized mutual information (NMI, mutual informationpost−transection/mutual informationbaseline) of voltage with adjacent electrode after combined white and gray matter disruption vs. sham controls (n = 10). Data represent mean ± s.e.m. [file Image4.TIF]

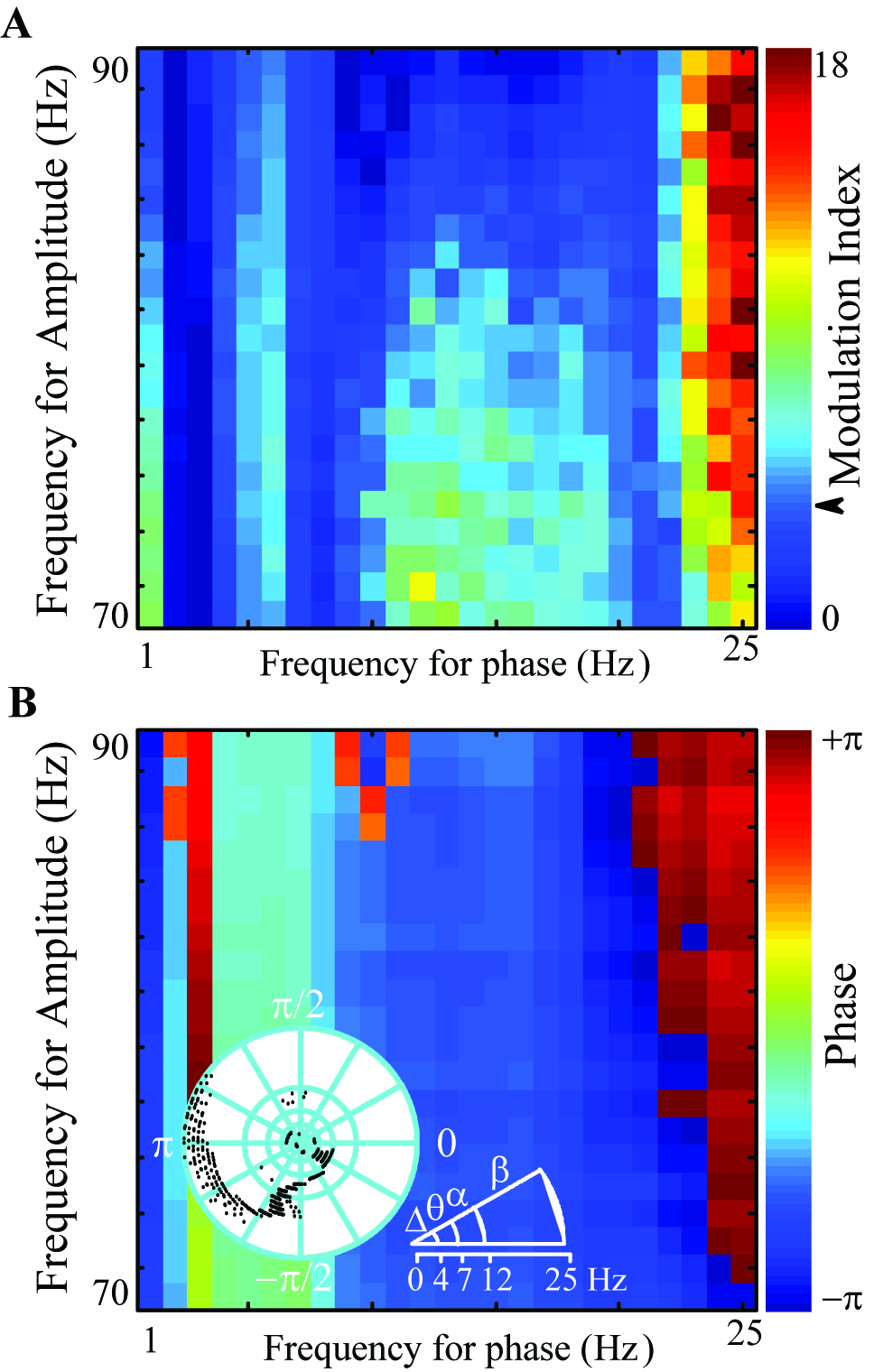

Supplement: Supplementary Figure 5 — Cortical explant displays phase-amplitude coupling ex vivo. Cross-frequency phase-amplitude coupling for high-γ amplitude showing modulation indices (A) and preferred phase with quantitation (B). Arrow indicates modulation index for p = 0.05. [file Image5.TIF]
