# Supplementary material for: Influence of White and Gray Matter Connections on Endogenous Human Cortical Oscillations
Source: Front Hum Neurosci. 2016 Jun 28;10:330. doi: 10.3389/fnhum.2016.00330 (PMC4923146; doi:10.3389/fnhum.2016.00330)
Supplement: Supplementary Table 1 — Demographic and clinical information. [file Table1.DOCX]

| **Table S1 \| Demographic and clinical information** | | | | | | | | | | | | |  | | |  | | | | |  |
| --- | --- | --- | --- | --- | --- | --- | --- | --- | --- | --- | --- | --- | --- | --- | --- | --- | --- | --- | --- | --- | --- |
| Subject | Sex | Age | Illness | Disease location | Duration of Illness | Anti-epileptic medications | Recording Location | Evaluation of  Recording Location Cortex | | Operation | | Experimental  Transections: | | | | | Dist. from pathology  (cm) | |  |  |  |
|  |  |  |  |  |  |  |  | Radiographic Examination* | Histological Examination^†^ |  | | | | Number of Electrodes | | |  | |  |  |  |
| 1 | M | 49 | Epilepsy | Left PFL | 2.25 years | CLP, LTG, LEV, TP | Left MFG | No abnormality | Normal | Left lesionectomy | | | | Both:2 | | | \| 3 \| \| --- \| | |  |  |  |
| 2 | M | 25 | Epilepsy | Left MTL | 7 years | CLP, LTG, LA | Left MTG | No abnormality | Not reviewed | Left AHC | | | | Both:2 | | | 3.6 | |  |  |  |
| 3 | M | 57 | Glioma | Left insula | 1 month | LEV | Left IFG | No abnormality | Not reviewed | Left lesionectomy | | | | Both:2 | | | 1.8 | |  |  |  |
| 4 | M | 71 | Glioma | Left MTL | 3 month | LEV | Right MTG | Normal Grey matter** | Not reviewed | Right temporal lobectomy | | | | Both:2 | | | 2 | |  |  |  |
| 5 | M | 56 | Epilepsy | Left MTL | 12 years | LTG, LEV, LA, TP | Left MTG | No abnormality | Normal | Left AHC | | | | Both:2 | | | 3.5 | |  |  |  |
| 6 | F | 22 | Epilepsy | Left TL | 20 years | TP, CBZ | Left MTG | No abnormality | Normal | Left temporal lobectomy | | | | Both:1 | | | 2.8 | |  |  |  |
| 7 | F | 51 | Epilepsy | Left MTL | 17 years | ZNA | Left MTG | No abnormality | Normal | Left AHC | | | | Both:1 | | | 3.1 | |  |  |  |
| 8 | M | 22 | Epilepsy | Left TL | 11 years | LTG, OX | Left MTG | No abnormality | Normal | Left temporal lobectomy | | | | Both:2 | | | 2.5 | |  |  |  |
| 9 | M | 58 | Epilepsy | Left MTL | 26 years | PH | Left MTG | No abnormality | Normal | Left AHC | | | | Both:1 | | | 3.2 | |  |  |  |
| 10 | M | 36 | Epilepsy | Right MTL | 26 years | TP, VA | Right MTG | No abnormality | Normal | | Right temporal lobectomy | | | | Both:2 | | | 3 | |  |  |

AHC, selective amygdalohippocampectomy; ATL, anterior temporal lobe; Both: white matter, grey matter and combined resections were performed; CBZ, carbamazepine; CLP, clonazepam; Dist, Estimated distance from pathology in centimeters; IFG, inferior frontal gyrus; LEV, levetiracetam; LA, lorazepam; LTG, lamotrigine; PFL, posterior frontal lobe; MFG, middle frontal gyrus; MTG, middle temporal gyrus; MTL, mesial temporal lobe; OX, oxcarbazepine; PH, phenytoin; TL, temporal lobe; TP, topiramate; ZNA, zonisamide; VA, valproate. *No abnormality indicates normal neocortex on Brain MRI when reviewed by a neuroradiologist. **Normal grey matter indicates normal grey signal on Brain MRI when reviewed by a neuroradiologist but the presence of T2 hyperintensity in deep white matter. ^†^Normal histological examination indicates normal laminar architecture, no evidence of dysplasia and no significant parenchymal changes, as reviewed by a neuropathologist.
