# Supplementary material for: Influence of White and Gray Matter Connections on Endogenous Human Cortical Oscillations
Source: Front Hum Neurosci. 2016 Jun 28;10:330. doi: 10.3389/fnhum.2016.00330 (PMC4923146; doi:10.3389/fnhum.2016.00330)
Supplement: Supplementary Table 2 — Change in power spectral density: Statistics Data. [file Table2.DOCX]

**Table S2 | Change in power spectral density**

| ANOVA | White vs. Sham | | | | | Grey vs. Sham | | |
| --- | --- | --- | --- | --- | --- | --- | --- | --- |
|  | DF | F | P |  | | DF | F | P |
| Subject | 6 | 157.71 | 8.76E-115 | |  | 5 | 9.78 | 6.97E-09 |
| Frequency | 89 | 2.03 | 8.43E-07 | |  | 89 | 0.95 | 0.605313 |
| Condition (Lesion vs. Sham) | 1 | 2603.61 | 1.74E-207 | |  | 1 | 58.57 | 1.23E-13 |
| Subject X Frequency | 534 | 0.97 | 0.629709 | |  | 445 | 0.96 | 0.657986 |
| Subject X Condition | 6 | 115.20 | 5.82E-93 | |  | 5 | 44.20 | 5.29E-37 |
| Frequency X Condition | 89 | 1.59 | 0.001078 | |  | 89 | 1.26 | 0.067481 |
| Within Groups | 534 |  |  | |  | 445 |  |  |
| Total | 1259 |  |  | |  | 1079 |  | |

| Change in Logarithmic PSD | | | | | | | Student’s *t*-test | | | | | | | | | | | | | | | | Binomial Probability of Reduced Power | | | | | | | |
| --- | --- | --- | --- | --- | --- | --- | --- | --- | --- | --- | --- | --- | --- | --- | --- | --- | --- | --- | --- | --- | --- | --- | --- | --- | --- | --- | --- | --- | --- | --- |
|  | Mean | | | Standard Error | | | White vs. Sham | | | | Grey vs. Sham | | | | | | White vs. Grey | | | | | | White (N=7) | | | | Grey (N=6) | | | |
| *f* | White | Grey | Sham | White | Grey | Sham | P | DF | T | P | | DF | | | T | P | | DF | | T | | N_r_ | | P | | N_r_ | | | P |  |
| δ | -1.13 | -0.06 | -0.06 | 0.28 | 0.13 | 0.06 | 0.020 | 12 | 3.17 | | 0.969 | | 11 | 0.001 | | | 0.019 | | 11 | | 3.25 | | 7 | | 0.008 | | 4 | 0.234 | | |
| θ | -1.08 | -0.23 | 0.03 | 0.31 | 0.17 | 0.04 | 0.027 | 12 | 3.00 | | 0.404 | | 11 | 1.37 | | | 0.089 | | 11 | | 2.37 | | 6 | | 0.055 | | 4 | 0.234 | | |
| α | -0.76 | -0.12 | 0.00 | 0.22 | 0.13 | 0.03 | 0.030 | 12 | 2.96 | | 0.653 | | 11 | 0.91 | | | 0.073 | | 11 | | 2.49 | | 6 | | 0.055 | | 2 | 0.234 | | |
| β | -0.74 | -0.11 | 0.09 | 0.19 | 0.13 | 0.05 | 0.012 | 12 | 3.45 | | 0.428 | | 11 | 1.33 | | | 0.056 | | 11 | | 2.64 | | 7 | | 0.008 | | 3 | 0.313 | | |
| γ | -0.73 | -0.21 | -0.02 | 0.15 | 0.11 | 0.05 | 0.005 | 12 | 3.91 | | 0.334 | | 11 | 1.52 | | | 0.038 | | 11 | | 2.86 | | 7 | | 0.008 | | 4 | 0.234 | | |

ANOVA, multiway analysis of variance; *f*, band frequency; DF, degrees of freedom; F, F-statistic, N, sample size; N_r_, number with reduced power; PSD, power spectral density; P, *p*-value ; T, T-statistic.
