# Supplementary material for: Influence of White and Gray Matter Connections on Endogenous Human Cortical Oscillations
Source: Front Hum Neurosci. 2016 Jun 28;10:330. doi: 10.3389/fnhum.2016.00330 (PMC4923146; doi:10.3389/fnhum.2016.00330)
Supplement: Supplementary Table 3 — Normalized mutual information of voltage shared with adjacent cortex: Statistics data. [file Table3.DOCX]

**Table S3 | Normalized Mutual Information of Voltage Shared With Adjacent Cortex**

|  | Normalized Mutual Information | | | Student’s T-test | | | | | | | | | | Binomial Probability of Increased NMI | | | | | |
| --- | --- | --- | --- | --- | --- | --- | --- | --- | --- | --- | --- | --- | --- | --- | --- | --- | --- | --- | --- |
|  | Mean ± standard error | | | White vs. Sham | | | Grey vs. Sham | | | White vs. Grey | | | | | White (N=11) | | Grey (N=10) | | |
| *f* | White | Grey | Sham | P | DF | T | P | DF | T | | P | DF | T | | N_r_ | P | | N_r_ | P |
| δ | 1.79 ± 0.44 | 1.65 ± 0.28 | 1.21 ± 0.10 | 0.445 | 19 | 1.26 | 0.337 | 18 | 1.47 | | 0.921 | 19 | 0.26 | | 8 | 0.081 | | 7 | 0.117 |
| θ | 1.97 ± 0.22 | 1.38 ± 0.11 | 1.08 ± 0.07 | 0.006 | 19 | 3.53 | 0.118 | 18 | 2.10 | | 0.074 | 19 | 2.34 | | 10 | 0.005 | | 8 | 0.044 |
| α | 1.98 ± 0.17 | 1.36 ± 0.11 | 1.07 ± 0.09 | 0.001 | 19 | 4.38 | 0.180 | 18 | 1.87 | | 0.022 | 19 | 2.93 | | 11 | 0.0005 | | 9 | 0.010 |
| β | 1.89 ± 0.30 | 1.56 ± 0.19 | 1.23 ± 0.13 | 0.149 | 19 | 1.97 | 0.388 | 18 | 1.37 | | 0.636 | 19 | 0.93 | | 10 | 0.005 | | 9 | 0.010 |
| γ | 2.50 ± 0.70 | 1.67 ± 0.21 | 0.96 ± 0.13 | 0.129 | 19 | 2.05 | 0.037 | 18 | 2.69 | | 0.529 | 19 | 1.11 | | 9 | 0.027 | | 10 | 0.001 |

Normalized Mutual Information (NMI) = Mutual information after lesion ÷ Mutual information at baseline. DF: degrees of freedom; *f*: band frequency; N: sample size; N_r_ number with increased NMI; P: *p*-value; T: T-statistic.
