# Supplementary material for: Influence of White and Gray Matter Connections on Endogenous Human Cortical Oscillations
Source: Front Hum Neurosci. 2016 Jun 28;10:330. doi: 10.3389/fnhum.2016.00330 (PMC4923146; doi:10.3389/fnhum.2016.00330)
Supplement: Supplementary Table 4 — Normalized mutual information of phase shared with adjacent cortex: Statistics data. [file Table4.DOCX]

**Table S4 | Normalized Mutual Information of Phase Shared With Adjacent Cortex**

| Normalized Mutual Information | | | | Student’s *t*-Test | | | | | | | | | Binomial Probability of Increased NMI | | | | |
| --- | --- | --- | --- | --- | --- | --- | --- | --- | --- | --- | --- | --- | --- | --- | --- | --- | --- |
|  | Mean ± standard error | | | White vs. Sham | | | Grey vs. Sham | | | White vs. Grey | | | | White (N=11) | | Grey (N=10) | |
| *f* | White | Grey | Sham | P | DF | T | P | DF | T | P | DF | T | | N_r_ | P | N_r_ | P |
| δ | 3.68 ± 1.58 | 1.55 ± 0.20 | 0.97 ± 0.09 | 0.269 | 18 | 1.63 | 0.101 | 17 | 2.22 | 0.432 | 19 | 1.29 | | 9 | 0.027 | 7 | 0.117 |
| θ | 2.61 ± 0.35 | 1.57 ± 0.22 | 1.29 ± 0.12 | 0.009 | 18 | 3.32 | 0.810 | 17 | 0.59 | 0.054 | 19 | 2.49 | | 10 | 0.005 | 9 | 0.010 |
| α | 2.89 ± 0.44 | 1.57 ± 0.17 | 1.18 ± 0.11 | 0.009 | 18 | 3.34 | 0.347 | 17 | 1.46 | 0.043 | 19 | 2.60 | | 11 | 0.0005 | 8 | 0.043 |
| β | 3.09 ± 0.55 | 1.45 ± 0.21 | 1.32 ± 0.10 | 0.026 | 18 | 2.86 | 0.988 | 17 | 1.22 | 0.042 | 19 | 2.61 | | 11 | 0.0005 | 7 | 0.117 |
| γ | 2.96 ±0.72 | 1.72 ± 0.25 | 1.26 ± 0.12 | 0.103 | 18 | 2.18 | 0.470 | 17 | 1.22 | 0.289 | 19 | 1.57 | | 9 | 0.027 | 8 | 0.043 |

Normalized Mutual Information = Mutual information after lesion ÷ Mutual information at baseline; DF, degrees of freedom; *f,* band frequency; N, sample size; N_r_, number with increased NM; P, *p*-value; T, T-statistic.
