# Supplementary material for: Influence of White and Gray Matter Connections on Endogenous Human Cortical Oscillations
Source: Front Hum Neurosci. 2016 Jun 28;10:330. doi: 10.3389/fnhum.2016.00330 (PMC4923146; doi:10.3389/fnhum.2016.00330)
Supplement: Supplementary Table 5 — Change in phase amplitude coupling of 1–25 Hz phases with 70–90 Hz amplitude: Statistics data. [file Table5.DOCX]

**Table S5 | Change in Phase Amplitude Coupling of 1-25 Hz phases with 70-90 Hz Amplitude**

| ANOVA | White vs. Sham | | | | Grey vs. Sham | | |
| --- | --- | --- | --- | --- | --- | --- | --- |
|  | DF | F | P |  | DF | F | P |
| Subject | 5 | 53.21 | 7.45E-29 |  | 5 | 34.11 | 1.49E-21 |
| Frequency of phase | 24 | 1.012 | 0.456382 |  | 24 | 1.54 | 0.067816 |
| Condition (Lesion vs. Sham) | 1 | 122.73 | 4.46E-20 |  | 1 | 217.52 | 1.02E-28 |
| Subject X Frequency | 120 | 1.02 | 0.454311 |  | 120 | 0.99 | 0.525716 |
| Subject X Condition | 5 | 101.19 | 2.40E-41 |  | 5 | 52.85 | 9.85E-29 |
| Frequency X Condition | 24 | 1.12 | 0.334741 |  | 24 | 2.26 | 0.002097 |
| Within Groups | 120 |  |  |  | 120 |  |  |
| Total | 299 |  |  |  | 299 |  | |

ANOVA, multiway analysis of variance; DF, degrees of freedom; F, F-statistic; P, *p*-value.
