# Supplementary material for: Influence of White and Gray Matter Connections on Endogenous Human Cortical Oscillations
Source: Front Hum Neurosci. 2016 Jun 28;10:330. doi: 10.3389/fnhum.2016.00330 (PMC4923146; doi:10.3389/fnhum.2016.00330)
Supplement: Supplementary Table 6 — Power spectral density after functional and physical disconnection from cortex. [file Table6.DOCX]

**Table S6 | Power Spectral Density after Functional and Physical Disconnection from Cortex**

|  | Logarithmic PSD | | | | | | | |
| --- | --- | --- | --- | --- | --- | --- | --- | --- |
|  | Mean |  |  |  | Standard Error | | | |
| *f* | Baseline | Complete | Explant | Noise | Baseline | Complete | Explant | Noise |
| δ | 8.46 | 3.00 | 4.83 | -3.20 | 0.46 | 0.55 | 1.04 | 0.56 |
| θ | 7.37 | 2.72 | 3.38 | -4.19 | 0.43 | 0.61 | 0.92 | 0.47 |
| α | 6.67 | 2.42 | 2.46 | -4.60 | 0.42 | 0.56 | 1.03 | 0.42 |
| β | 6.35 | 2.18 | 1.70 | -5.02 | 0.51 | 0.70 | 1.21 | 0.35 |
| γ | 5.64 | 1.92 | 0.67 | -5.29 | 0.58 | 0.66 | 1.25 | 0.36 |

|  | Students *t*-test | | | | | | | | | | | | | | |  |  |  |
| --- | --- | --- | --- | --- | --- | --- | --- | --- | --- | --- | --- | --- | --- | --- | --- | --- | --- | --- |
|  | Baseline vs. Noise | | | Both vs. Noise | | | Explant vs. Noise | | | Baseline vs. Explant | | | Both vs. Explant | | | Baseline vs. Both | | |
| *f* | P | DF | T | P | DF | T | P | DF | T | P | DF | T | P | DF | T | P | DF | T |
| δ | 5.00E-15 | 41 | 12.38 | 2.63E-11 | 41 | 9.34 | 0.0008 | 39 | 3.94 | 0.0504 | 10 | 2.75 | 0.1950 | 10 | 1.91 | 0.250 | 12 | 1.72 |
| θ | 2.22E-14 | 41 | 11.82 | 6.84E-09 | 41 | 7.56 | 0.0014 | 39 | 3.77 | 0.0414 | 10 | 2.86 | 0.1316 | 10 | 2.16 | 0.449 | 12 | 1.28 |
| α | 1.16E-13 | 41 | 11.21 | 3.71E-09 | 41 | 7.75 | 0.0053 | 39 | 3.29 | 0.0557 | 10 | 2.69 | 0.1378 | 10 | 2.14 | 0.438 | 12 | 1.30 |
| β | 1.20E-09 | 41 | 8.10 | 1.21E-06 | 41 | 5.97 | 0.0190 | 39 | 2.81 | 0.0713 | 10 | 2.54 | 0.1720 | 10 | 1.99 | 0.521 | 12 | 1.15 |
| γ | 2.09E-08 | 41 | 7.21 | 5.53E-07 | 41 | 6.20 | 0.0342 | 39 | 2.58 | 0.0594 | 10 | 2.65 | 0.1272 | 10 | 2.19 | 0.593 | 12 | 1.02 |

DF, degrees of freedom; *f,* frequency band; T, T-statistic; P, *p*-value; PSD, power spectral density.
