# Supplementary material for: Influence of White and Gray Matter Connections on Endogenous Human Cortical Oscillations
Source: Front Hum Neurosci. 2016 Jun 28;10:330. doi: 10.3389/fnhum.2016.00330 (PMC4923146; doi:10.3389/fnhum.2016.00330)
Supplement: Supplementary Table 7 — phase amplitude coupling after functional disconnection: Statistics data. [file Table7.DOCX]

**Table S7 | Phase Amplitude Coupling after Functional Disconnection**

| ANOVA | Complete vs. Baseline | | | | | Sham vs. Baseline | | |
| --- | --- | --- | --- | --- | --- | --- | --- | --- |
|  | DF | F | | P |  | DF | F | P |
| Subject | 5 | 61.21 | 2.13E-31 | |  | 5 | 515.17 | 0 |
| Frequency of phase | 24 | 2.17 | 0.003324 | |  | 24 | 11.13 | 1.44E-20 |
| Condition (Lesion vs. Baseline) | 1 | 215.28 | 1.52E-28 | |  | 1 | 77.72 | 1.11E-14 |
| Subject X Frequency | 120 | 0.81 | 0.874774 | |  | 120 | 8.81 | 2.94E-28 |
| Subject X Condition | 5 | 86.94 | 3.22E-38 | |  | 5 | 17.06 | 1.01E-12 |
| Frequency X Condition | 24 | 2.90 | 7.04E-05 | |  | 24 | 0.93 | 0.558911 |
| Within Groups | 120 |  |  | |  | 120 |  |  |
| Total | 299 |  |  | |  | 299 |  | |

ANOVA, multiway analysis of variance; DF, degrees of freedom; F, F-statistic; P, *p*-value.
